# A neural model of hierarchical reinforcement learning

**DOI:** 10.1371/journal.pone.0180234

**Published:** 2017-07-06

**Authors:** Daniel Rasmussen, Aaron Voelker, Chris Eliasmith

**Affiliations:** 1 Applied Brain Research, Inc., Waterloo, ON, Canada; 2 Centre for Theoretical Neuroscience, University of Waterloo, Waterloo, ON, Canada; Georgia State University, UNITED STATES

## Abstract

We develop a novel, biologically detailed neural model of reinforcement learning (RL) processes in the brain. This model incorporates a broad range of biological features that pose challenges to neural RL, such as temporally extended action sequences, continuous environments involving unknown time delays, and noisy/imprecise computations. Most significantly, we expand the model into the realm of hierarchical reinforcement learning (HRL), which divides the RL process into a hierarchy of actions at different levels of abstraction. Here we implement all the major components of HRL in a neural model that captures a variety of known anatomical and physiological properties of the brain. We demonstrate the performance of the model in a range of different environments, in order to emphasize the aim of understanding the brain’s general reinforcement learning ability. These results show that the model compares well to previous modelling work and demonstrates improved performance as a result of its hierarchical ability. We also show that the model’s behaviour is consistent with available data on human hierarchical RL, and generate several novel predictions.

## 1 Introduction

One of the basic problems brains must solve is how to achieve good outcomes in unfamiliar environments. A rat trying to navigate a maze, a bird trying to decide where to forage, or a human trying to impress a new boss—all are faced with the problems of being in an unknown environment, having no clear indication of how to achieve their target, and executing a potentially lengthy sequence of decisions in order to achieve their goals.

Reinforcement learning (RL; [1]) is a computational approach that aims to address this type of problem. Specifically, RL seeks to understand how an agent can perform well when it begins with minimal knowledge of its environment and receives only sparse feedback to guide its actions. RL is also one of the best examples of cross-fertilization between computational theories and experimental investigation of the brain. RL theories have been used to provide new explanations for empirical data, and neurophysiological data has inspired the development of new computational algorithms [2].

This cross-fertilization is realized even more explicitly in computational neural modelling—the practice of building detailed mechanistic models that recreate neural function. These models can be used to explain how the abstract computations of reinforcement learning could be carried out by real brains. One way to succinctly summarize the motivation for such work is as follows:

1. Brains must solve reinforcement learning style problems somehow, as evidenced by their impressive behavioural performance
2. There are algorithms in RL that provide powerful methods for solving such problems computationally
3. If modellers can show how those methods can be implemented in neural systems, we then have a hypothesis for how the brain could achieve those same solutions

Of course there are challenges to this idealized approach. One of the most critical is hinted at in point 2, with the question being “just how powerful are these algorithms?” Reinforcement learning has a 30+ year history in computer science; many different techniques have been developed, all with their own strengths and weaknesses. Thus it is quite important which computational account from point 2 a modeller chooses to implement in point 3, as the resulting neural theory will have similar strengths and weaknesses to the computational theory. Unfortunately, much neural modelling work has been based on some of the earliest computational theories, and we therefore know that the proposed neural system will have the same limitations as those theories. For example, many models can only learn to maximize immediate reward (known as “associative RL”)—they cannot learn to execute a series of unrewarded actions in order to achieve a larger reward in the future [3–8]. This is not to say that there are not important insights to be gained from such models, for example with respect to the mechanisms of dopamine modulated plasticity. But from a functional perspective, we know that these mechanisms are not enough to support the temporally extended decision making behaviour observed in humans and other animals.

Another challenge arises when incorporating features of biological complexity into idealized computational algorithms. This is almost never a straightforward translation process, and can require fundamental changes to the underlying algorithm; that is why constructing biologically detailed neural models is important, if we want to understand the relationship between idealized algorithms and the imperfect computational system of the brain [9]. For example, many RL algorithms instantiated in neural models assume that space and time are divided into discrete steps (e.g., [10–12]), that computations are completely accurate and noiseless (e.g., [13]), or that functions have perfect access to information from other areas of the model or previous points in time (e.g., [14, 15]). When we increase the biological realism of these models it is often necessary to modify their implementation, such as increasing the number of neurons to counteract the less precise output of each neuron. However, this can lead to an implausibly/impractically large number of neurons (e.g., a significant portion, or more, of all neurons that exist in the brain or some modelled subregion [16]). Thus we may need to adapt the algorithm itself, for example to reduce the required precision. Or in other cases we may need to introduce entirely new components to the algorithm, such as a memory system to preserve information from previous points in time. It is not a guarantee that this process will result in success; some algorithms have crucial assumptions that are simply not possible to translate into a biologically detailed implementation. This means that if we want to know whether an abstract RL algorithm is a plausible hypothesis for RL processing in the brain, demonstrating a successful biological neural implementation of that algorithm is an important step.

A further difficulty for many models is scaling up to complex problem spaces. While even the simplest models may be guaranteed to find the correct solution in some domain, as the problem space becomes more complex it can take impractical amounts of time or resources to find that solution. Hierarchical reinforcement learning (HRL; [17]) is a computational approach aimed at addressing this difficulty. The basic idea behind HRL is to decompose the overall RL task into subtasks, whose solutions can be learned more tractably. Those subtask solutions represent abstract actions, such that if the agent executes that action it will carry out the subtask. The agent then needs to learn how to select between different abstract and primitive actions in order to complete the overall task. This decomposition has a number of benefits (discussed in more detail in Section 2.2), allowing reinforcement learning to scale to more complex problems. Thus HRL is an intriguing candidate as an account for reinforcement learning processes in the brain, as it would scale better to the complex problems faced in the real world. However, in order to pursue that hypothesis we need to address the above issue: can this theory be adapted so as to be implemented in a biologically plausible neural model? This has not been demonstrated by any previous model (although there have been promising first steps [18]), so it is as yet unclear whether HRL might be a plausible account of hierarchical learning in the brain.

In this work we construct such a model, which we call the Neural HRL (NHRL) model. This is the first neural model to implement the computational processes of HRL. It can operate in environments that are continuous in both time and space, and that involve lengthy, variable, and unknown time delays. In addition, it can operate within the constraints of a realistic neural environment, such as local information transfer, heterogeneous components, and imprecise computations. We begin by discussing the underlying theories of HRL and neural modelling in more detail, as well as briefly reviewing previous modelling work in this area. We then present the NHRL model, followed by results on several different tasks. We conclude with a discussion of the open questions highlighted by this work, as well as some of the predictions arising from the model.

## 2 Background

### 2.1 Reinforcement learning

The basic problem to be solved by reinforcement learning is this: given the current state of the world, what is the best action to take? Most commonly, the “world” is described formally in the language of Markov Decision Processes (MDPs; [19]), where the task has some state space *S*, available actions *A*, transition function *P*(*s*, *a*, *s*′) (which describes how the agent will move through the state space given a current state *s* and selected action *a*), and reward function *R*(*s*, *a*) (which describes the feedback the agent will receive after selecting action *a* in state *s*). In this framework, the “best action” is the one that maximizes the expected long term reward received by the agent.

The value of taking action *a* in state *s* is defined as the total reward received after selecting *a* and then continuing on into the future. This can be expressed recursively through the standard Bellman equation as

$$\begin{array}{c} Q\left(s,a\right)=R\left(s,a\right)+\gamma \sum_{s^{\prime }}P\left(s,a,s^{\prime }\right)Q\left(s^{\prime },\pi \left(s^{\prime }\right)\right) \end{array}$$ (1)

where *π*(*s*) is the agent’s policy, indicating the action it will select in the given state. The first term corresponds to the immediate reward received for picking action *a*, and the second term corresponds to the expected future reward (the *Q* value of the policy’s action in the next state, scaled by the probability of reaching that state). *γ* is a discounting factor, which is necessary to prevent the expected values from going to infinity (since the agent will be continuously accumulating more reward).

Temporal difference (TD) learning is a method for learning those *Q* values in an environment where the transition and reward functions are unknown, and can only be sampled by exploring the environment [1]. It accomplishes this by taking advantage of the fact that a *Q* value is essentially a prediction, which can be compared against observed data. Specifically, the *Q* values are updated according to

$$\begin{array}{c} \Delta Q\left(s,a\right)=\alpha [r+\gamma Q\left(s^{\prime },a^{\prime }\right)-Q\left(s,a\right)] \end{array}$$ (2)

where *α* is a learning rate parameter. The value within the brackets is referred to as the temporal difference/prediction error. Note that here the functions *R*(*s*, *a*), *P*(*s*, *a*, *s*′), and *π*(*s*′) have been replaced by the samples *r*, *s*′, and *a*′, respectively. Those samples allow us to approximate the value of action *a*, which we compare to the predicted value *Q*(*s*, *a*) in order to compute the update to the prediction. The agent can then determine a policy based on those *Q* values, usually by selecting the highest valued action in each state (with occasional random exploration).

### 2.2 Hierarchical reinforcement learning

As mentioned, HRL attempts to improve the practical applicability of the basic RL theory outlined above, through the addition of hierarchical processing. There are several different approaches to HRL ([20–22]; see [17, 23] for a review). In this section we try to describe HRL as generally as possible, without reference to the detailed distinctions of these approaches. However, we draw most heavily on the options framework of [21].

The central idea of hierarchical reinforcement learning (HRL) is the notion of an abstract action (e.g., “options” in [21]). Abstract actions work like shortcuts, encapsulating whole sequences of decisions (the basic actions that actually carry out the abstract action) in a single choice. This framework is hierarchical because abstract actions can themselves be components in other abstract actions. For example, imagine a robotic agent navigating around a house. Basic actions might include “turn left”, “turn right”, and “move forward”. An abstract action might be “go to the kitchen”. Selecting that action will activate a subpolicy designed to take the agent from wherever it currently is to the kitchen via a sequence of basic actions. And, hierarchically, “go to the kitchen” could itself be one of the actions in a more abstract policy for “make dinner”.

The incorporation of abstract actions helps to address the challenges faced by RL in a number of different ways [24]. Perhaps the most basic is that it speeds reward propagation throughout the task. Returning to our example, imagine an agent starting in the bedroom and trying to learn how to navigate to the refridgerator. A long sequence of basic actions will be required in order to complete the task, thus the agent is faced with a challenging credit assignment problem when trying to decide what the best action is in the bedroom. But suppose the agent selects the “go to the kitchen” action, and then a few basic actions to take it from the centre of the kitchen to the refridgerator. Reward information can then propagate directly from the kitchen to wherever the agent selected the “go to the kitchen” action. The agent can quickly learn whether selecting “go to the kitchen” was a good choice in the bedroom, even though there were many basic actions separating the decision from the eventual outcome. In other words, the complexity of learning the value of an abstract action is relatively independent of the length of the actual decision path that action will invoke.

Another important advantage of HRL is that it promotes better exploration. One of the weaknesses of RL is that learning tends to begin with a long period of random action selection, or “flailing”. This results in a kind of Brownian motion, where the agent moves around in a limited area rather than exploring throughout the state space. One can imagine that if our refrigerator-seeking agent begins selecting random basic actions in the bedroom, it will spend a long time wandering around the bedroom before it gets anywhere close to the kitchen. But if the agent randomly selects the “go to the dining room” action, that will take it to a significantly different area of the state space. Thus the agent’s random exploration is going to result in a much broader coverage of the search space, and therefore is more likely to bring it within proximity of the goal.

Note that both the above advantages are dependent on the quality of the abstract actions; including unhelpful actions, such as “go to the roof”, can actually make the problem more difficult for the agent [25]. Even if the abstract actions are useful, they increase the complexity of the problem by expanding the action space, so they must provide benefits that outweigh those innate costs [26]. The question of how to discover useful abstract actions is an important and open problem in the computational study of HRL, but beyond the scope of this paper (we will return to this in Section 6).

A third advantage of HRL is that it lends itself to state abstraction. State abstraction is the process of ignoring parts of the state that are irrelevant to the current task, thus reducing the size of the state space. In HRL it is possible to associate different state abstractions with the different abstract actions. For example, suppose the agent is trying to learn a subpolicy to get to the doorway of the bedroom. In that case it does not really matter what is going on anywhere else in the house, so that subpolicy can be learned based only on the parts of the state pertaining to the bedroom. This will make it much easier to learn that subpolicy. Again, the question of how to come up with useful state abstractions is nontrivial (e.g., how does the agent know which aspects of the state are associated with the bedroom, or which it is safe to ignore?). However, this question is more easily addressed in the hierarchical case, as the abstract actions are restricted to limited parts of the task by design. Without HRL the agent must try to find a state abstraction that works for the whole task, which is likely to be more difficult to find and also likely to eliminate a smaller portion of the state space.

The use of transfer learning in HRL is a similar case, in that it is not an intrinsic benefit of HRL but is made easier by the hierarchical framework. Transfer learning is the process of using knowledge gained in a previous task to aid performance in a new task [27]. While this is possible in other RL frameworks, it is made much easier by the use of HRL. One of the main challenges of transfer learning is trying to separate the knowledge that can be reused from the knowledge specific to the previous task. In HRL, knowledge is already divided into natural modular chunks—the abstract actions. The abstract actions tend to be self-contained, general, and well-defined, making them perfect components for transfer learning. For example, it is easy to see how the “go to the kitchen” action could be reused for navigating to the refrigerator, the oven, the sink, and so on. Once that subpolicy has been learned once, it can be used as an abstract action in these new tasks, thereby conferring all of the benefits described above.

#### 2.2.1 Adding temporal delays to Markov Decision Processes

When an agent selects a basic action, the result of that action can be observed in the next timestep (by the definition of MDPs). But abstract actions are not completed in a single timestep—there is some time interval that elapses while the subpolicy is executing the underlying basic actions, and only at the end of that delay period can the results of that abstract action be observed. Thus we need to add the notion of temporal delays into the MDP-based reinforcement learning framework.

This can be achieved using the language of Semi-Markov Decision Processes (SMDPs; [28]). In an SMDP environment the value of selecting action *a* in state *s* is equal to the summed reward received across the delay period, plus the action value in the resulting state, all discounted across the length of the delay period *τ*. The prediction error equation (Eq (2)) can be re-expressed as

$$\begin{array}{c} \Delta Q\left(s,a\right)=\alpha [\sum_{t=0}^{\tau -1}\gamma^{t}r_{t}+\gamma^{\tau }Q\left(s^{\prime },a^{\prime }\right)-Q\left(s,a\right)] \end{array}$$ (3)

This allows the agent to learn the value of both primitive and abstract actions (we can think of primitive actions as a special kind of abstract action that always terminate after one step).

Note that this is from the perspective of a single layer in the hierarchy. The hierarchical system must also learn the subpolicies associated with each abstract action, if they are not given. For example, in addition to learning how to sequence the “go to doorway” and “go to kitchen” actions to complete the overall task, the agent must also learn the sub-policy that will carry out the “go to the doorway” action. However, because everything has been reframed in terms of abstract actions (as in Eq (3)), there is no qualitative distinction between the policy that carries out the overall task and the sub-policy that carries out an abstract action. We can think of them both as a task with some goal, in which the agent must learn to select between the available actions in order to maximize reward. Thus learning the subpolicy can be accomplished in the same way as Eq (3), but in the state and action space of the subpolicy. Note that this also requires rewards for the subpolicy, which may be distinct from the task rewards (referred to as “pseudoreward”). This will be discussed in more detail when we look at specific implementations of HRL in this model. It is also useful to point out that these learning processes can be occurring simultaneously as the agent moves through the environment, allowing the system to learn across the different levels of abstraction.

With that basic framework in place there are still many different ways to implement HRL, based on issues such as how the hierarchy of actions is structured and how the prediction error is calculated and applied. Different HRL theories are defined by their choices on these issues. We draw a more detailed comparison to these theories in the supplementary material (S1 File).

### 2.3 Neural engineering framework

The previous sections have outlined the computations involved in RL/HRL. The Neural Engineering Framework (NEF; [29]) is the tool we use to bridge the gap between those computational descriptions and a neural implementation. A full overview of the NEF is beyond the scope of this article; here we will focus on the aspects most relevant to this work, and refer the interested reader to [29] for more detail.

A central feature of the NEF is the ability to translate computational variables (such as states or action values) into neural activity, and decode neural activity back into computational variables the modeller can analyze. This is accomplished via a distributed, population-based representation scheme. Specifically, encoding a vector *x* into the activity of neuron *i* is accomplished via

$$\begin{array}{c} a_{i}\left(x\right)=G_{i}\left[\alpha_{i}e_{i}x+J_{i}^{\mathrm{bias}}\right] \end{array}$$ (4)

which describes the activity *a*_*i*_ of neuron *i* as a function of its input current. *G*_*i*_ is a function representing the nonlinear neuron characteristics. It takes a current as input (the value within the brackets), and uses a model of neuron behaviour to output firing activity. In this work we use the leaky integrate and fire (LIF; [30]) neuron model, which strikes a balance between biological detail and computational simplicity. One of the nice features of the LIF model is that it can output either an overall firing rate or individual spikes; we will show that either can be used in this model in the results section (see Section 5.2 for a rate-based implementation of the model and Section 5.3 for a spiking implementation).

The variables *α*_*i*_, $J_{i}^{\mathrm{bias}}$, and *e*_*i*_ are the parameters of neuron *i*. The parameters *α*_*i*_ and $J_{i}^{\mathrm{bias}}$ do not directly play a role in the encoding of information, but rather are used to provide variability in the firing characteristics of neurons. They are chosen randomly from ranges that give biologically plausible response curves; this allows the modeller to capture the heterogeneity observed in biological neurons. The parameter *e*_*i*_ is a vector representing the neuron’s preferred stimulus. Specifically, the dot product between *e*_*i*_ and the input *x* (i.e., their similarity) drives a particular cell, so *e*_*i*_ defines which types of inputs will cause the neuron to respond most strongly.

Decoding is accomplished via a linear least squares procedure [31], which is used to find a linear weighting over the neural response functions (*a*_*i*_) that best approximates a target function. This allows for very efficient, analytic computation of connection weights for fixed transformations, such as the steps involved in computing a temporal difference error.

However, in some cases the required transformation is not known ahead of time; for example, in reinforcement learning the *Q* function is not known until the agent actually starts exploring its environment. In those cases the weights need to be learned online. In our work we use the Prescribed Error Sensitivity rule (PES; [32]). This is described by the formula:

$$\begin{array}{c} \Delta \omega_{ij}=\kappa \alpha_{j}e_{j}Ea_{i}\left(x\right) \end{array}$$ (5)

where *κ* is a learning rate, *α*_*j*_ and *e*_*j*_ are parameters of the postsynaptic neuron (described above), *E* is an error signal, and *a*_*i*_(*x*) is the activity of the presynaptic neuron. This is an error modulated local learning rule, which we can think of as performing gradient descent on the output weights of the presynaptic neuron based on the error signal. In other words, this learning rule will cause the transformation calculated by the connection weights to be adjusted in the direction of *E*. For example, if the output of the *a* population represents *Q* values, and *E* is the TD error, this will cause the *Q* values to be adjusted as in Eq (2).

The actual construction and simulation of NEF models is carried out by a software suite called Nengo [33, 34]. Nengo provides a high-level functional perspective to the modeller, and implements the NEF mathematics behind the scenes. Nengo is an open-source project (http://www.nengo.ca), and all the code used to construct the model we present here is available at https://github.com/drasmuss/nhrlmodel.

## 3 Previous neural modelling

We begin by discussing standard (non-hierarchical) reinforcement learning models, as several new developments incorporated in the NHRL model also address open issues there. We then discuss the much more sparsely populated domain of neural HRL models.

### 3.1 Neural models of reinforcement learning

As the move is made to more biologically plausible models, often there is a trade-off between biological detail and functionality. Purely computational systems have the option to ignore some of the challenges faced by real physical systems, such as limited precision, capacity, and local information transfer. Thus when biologically based models add these extra constraints, it is often necessary to simplify the computations they are performing.

One simplification common to these models is that they restrict themselves to “associative reinforcement learning”. In associative RL the agent does not consider the future impact of its actions (i.e., the value of the subsequent state), it just tries to pick whichever action will result in the largest immediate reward. That is, instead of representing the state-action value as

$$\begin{array}{c} Q\left(s,a\right)=R\left(s,a\right)+\gamma \sum_{s^{\prime }}P\left(s,a,s^{\prime }\right)Q\left(s^{\prime },\pi \left(s^{\prime }\right)\right) \end{array}$$

it is simply

$$\begin{array}{c} Q(s,a)=R(s,a) \end{array}$$ (6)

This means that the associative RL update is Δ*Q*(*s*, *a*) = *α*[*r* − *Q*(*s*, *a*)] (compare to the TD update in Eq (2)). The majority of work in biological RL modelling has been on this type of problem [3–8]. In cases where the agent’s actions do not have any long term consequences beyond the immediate reward (such as a bandit task), associative RL is all that is needed to perform well on the task. However, imagine a task where the agent can choose between two actions. Action *A* gives a small negative reward, but allows the agent to select an action on its next step leading to a large positive reward. Action *B* gives a small positive reward and nothing else. An associative RL agent will always learn to choose action *B* over action *A*, even though it could achieve more reward in the long run by selecting *A*. Sacrificing short term losses for long term gains is one of the fundamental challenges of decision making (and motivations for advances such as TD learning), and if we want to be able to model that behaviour we need to go beyond associative RL.

As a sidenote, there is another class of models that compute the TD error outside the model and then feed it in as an input signal (e.g., [15, 35–37]). From an implementation perspective, learning can then be accomplished in the same way as associative RL, because the model only needs to pick the action in each state that will result in the highest immediate “reward”—the difference is that the “reward” in this case is the externally computed signal, rather than the environmental reward. From a behavioural perspective this does capture the behaviour of interest, and these models can address many important questions. However, if we are interested specifically in neural mechanisms for temporally extended learning, then such a system is solving an associative RL problem.

One approach to moving beyond associative RL is the use of eligibility traces. The basic idea of an eligibility trace is to add a slowly decaying representation of some signal of interest (such as recently visited states). Then, rather than just updating the state immediately preceding the prediction error, we update all the states leading up to that prediction error, weighted by the decaying eligibility trace. Thus a model can use essentially the same associative RL framework, but with the benefit of eligibility traces the model can learn a value for the states leading up to the reward, rather than just the state with immediate reward. Returning to the above example, when the agent receives a large positive reward for the later action, the eligibility trace for *A* will still be elevated, and some of that reward information will be associated with *A*. [38] and [39] are examples of this approach, combining associative RL with eligibility traces.

However, there are important limitations to such an approach. The main one is that at some point the eligibility trace will have decayed to a point where the model cannot distinguish it from zero, which will mark the limit of how far away from the goal the agent can make effective decisions. Note that in purely computational systems (with perfect precision in the represented values) there is no such limit, since the eligibility trace can be tracked indefinitely. However, in a realistic, noisy, imprecise neural model there will be a fixed limit to the effective range of any eligibility trace. Another limitation is that eligibility traces perform indiscriminate credit assignment, meaning that they will associate a reward with any temporally preceding actions. This is generally not a problem for standard RL, but if we introduce structure into the action space (e.g., through the addition of hierarchical actions), wherein temporally adjacent actions may need to be treated qualitatively differently, then eligibility traces do not lend themselves to that type of processing.

One of the most advanced neural reinforcement learning models is the work described by Potjans et al. in [12]. Their model also makes use of eligibility traces, but not in the same way as above. Rather than using eligibility traces to replace the TD error calculation, this model uses eligibility traces to compute the TD error. Two eligibility traces with different time constants are used to compute the change in state value, which when combined with reward is sufficient to compute a prediction error. However, this still imposes a fixed time period during which the TD error can be computed. If the TD update does not occur within the time window dictated by the decay rate of the slow trace, then it will not be possible to compute a meaningful TD error.

A fixed window is feasible in an MDP framework because rewards and state transitions all occur on a fixed schedule, which we can assume falls within that window. But in an SMDP environment, i.e., one where actions do not terminate on a fixed schedule, the learning update may need to be performed after 100ms or after 10s; the system does not know the delay ahead of time, so it cannot be hard coded into the eligibility traces. This is an even greater problem in the case of hierarchical RL, as the state may be changing during the delay period; in that case the value trace from the beginning of the delay period will have long since been replaced by intermediate values by the end of the delay period. Thus while the model in [12] is a solution to the basic TD RL problem, we will not be able to use this method to implement an SMDP RL algorithm (such as HRL). We compare the performance of the model from [12] to the NHRL model in Section 5.1.

### 3.2 Hierarchical reinforcement learning

In recent years there has been significant interest in neural correlates of hierarchical reinforcement learning [25, 40–44]. However, in contrast to standard RL, there has been almost no previous work on recreating the computational theory of hierarchical reinforcement learning in a neural model.

The work of [40] develops a proposal as to how the actor critic architecture could be modified in order to implement the options framework of HRL. The implementation itself is purely computational, with no neural components, but [40] includes a detailed discussion of how that proposal could map onto neural components in theory. The model in [43] is similar, in that the implementation itself is non-neural, but the model is used to gain interesting insights into neural data on hierarchical processing in the brain.

In [18] the authors extend a previous working memory model [10] to a hierarchical architecture. However, this model is not intended to be an implementation of the computational theory of HRL. It is designed specifically for tasks with hierarchical spatial structure, not hierarchical temporal structure. Although this is a component of HRL, it does not address the problem of temporally extended abstract actions that is at the core of HRL processing (e.g., Eq (3)); we can think of this model as performing “associative HRL”. This is not intended as a critique of the model in [18], but rather to show that the problem it addresses is not the same as the one we focus on in the work presented here. However, for the sake of comparison we contrast their results with the NHRL model in Section 5.3.

## 4 Model description

**Fig 1:**
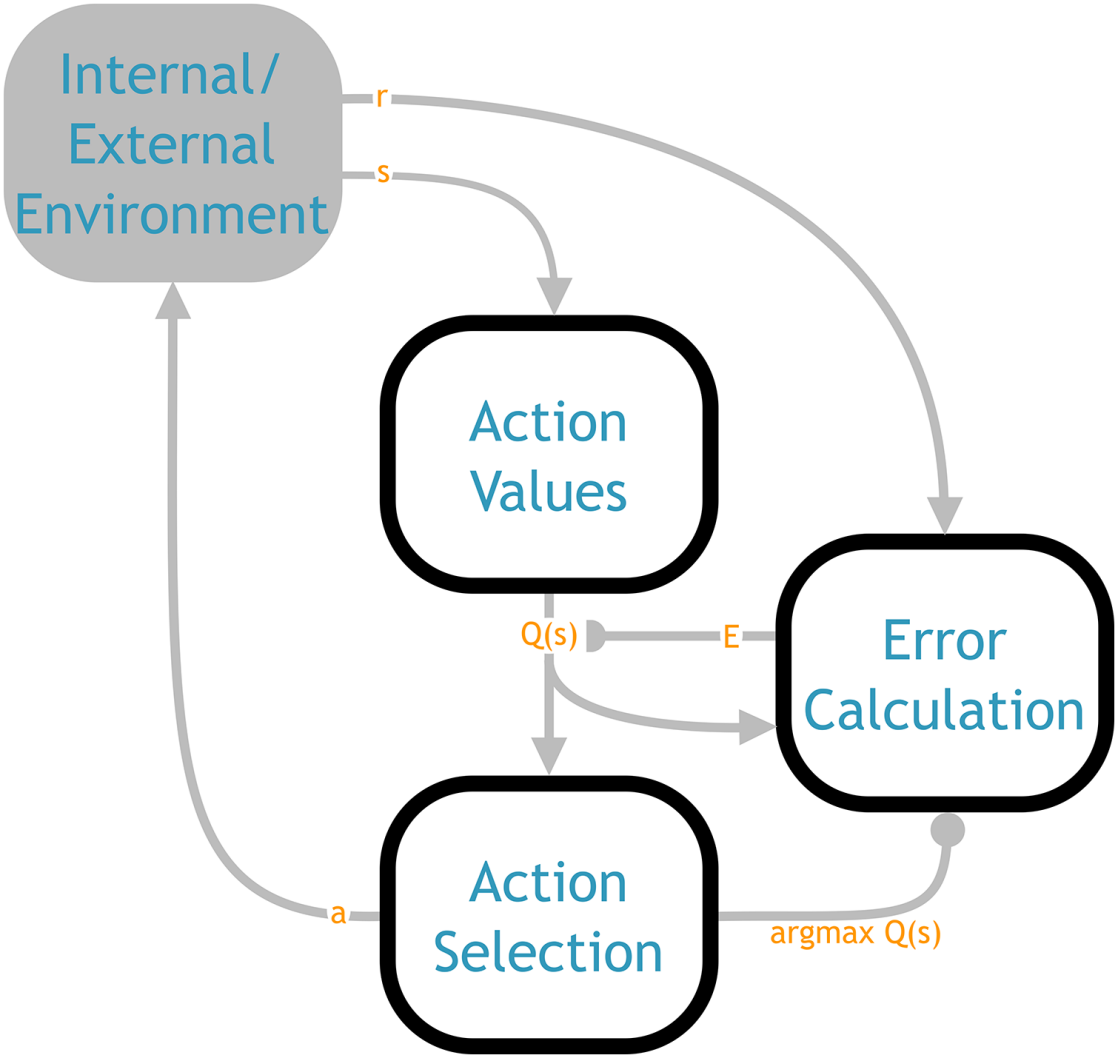
Overall architecture of the NHRL model, showing the three main components and the functional values flowing between them. The action values component computes the *Q* values given the state from the environment (see Fig 2 for more detail). The action selection component determines the highest valued action, and sends the action itself to the environment and the identity of the selected action to the error calculation component (see Fig 3 for more detail). The error calculation component uses the *Q* values and environmental reward to calculate the TD error, which it uses to update the *Q* function in the action values component (see Fig 4 for more detail). Triangular arrowheads indicate a vector value connection, semicircle indicates a modulatory connection that drives learning, and circular arrowheads denote an inhibitory connection. See Fig 6 for a mapping of this abstract architecture onto neuroanatomical structures.

We have divided the structure of the NHRL model into three main components, which we term action values, action selection, and error calculation (shown in Fig 1). We begin by discussing each of these components in turn, and show how they implement their respective aspects of reinforcement learning. Together these components form a flat, non-hierarchical system. Although the underlying design decisions were made with the needs of a hierarchical system in mind (e.g., SMDP processing), this aspect of the model can be understood without any reference to HRL. After the basic model is presented, we then show how these elements can be composed into a hierarchical structure.

We will focus here largely on the model’s higher level computations. For more detail on the neural implementation, see [45].

### 4.1 Task environment

We present the model largely without reference to any particular task or environment. The model is designed as a generic reinforcement learning system, thus the implementation is not based on any specific task. Rather, the components are constructed in a general fashion, so that this same model can be applied in many different environments.

To this end, the agent treats the environment largely as a black box. Its only insight into the environment is a real-valued vector that the environment makes available—the state. This state is assumed to be continuous in both time and space. The only way for the agent to interact with the environment is by outputting a real-valued vector representing an action. The agent assumes that the action space is given (i.e., the agent knows what possibilities it has to choose between), but it does not know anything about the effect or value of those actions. The environment takes the action output from the agent, updates the state through some unknown internal mechanisms, and delivers the new state to the agent (in continuous time). The only goal-based feedback the agent receives is a scalar value from the environment, the reward, and it will seek to select actions that maximize the long term cumulative value of that reward.

Note that we use “environment” here in a general sense, to refer to all of the state and reward generating processes occurring outside this model. This could include processes internal to the agent; for example, visual processing, memory systems, somatosensory feedback, and so on. The NHRL model makes no assumptions about the source of its inputs.

### 4.2 Action values component

The first aspect of the model is the representation of state-action values—that is, a neural representation of the *Q* function. The central feature of this component is a single population of neurons. These neurons take the continuous environmental state *s* as input, and output an *n*-dimensional vector (*n* is the number of available actions, |*A*|) where each element represents the *Q* value of that action in the current state. We will refer to this vector as *Q*(*s*), i.e., $Q\left(s\right)=[Q\left(s,a_{1}\right),Q\left(s,a_{2}\right),\cdots ,Q\left(s,a_{n}\right)]$. Note that when we say that the population outputs a vector, we refer to the value decoded using the techniques of the NEF. The actual output is a vector of neural activities with length *m*, where *m* is the number of neurons in the population, but it is generally more useful to refer to the represented value.

We need a brief aside here to mention what we mean by *s* and *s*′, in the context of a system operating in continuous time and space. That is, *s*(*t*) is a continuously changing signal, it is not divided up into previous and current states. By *s* we mean the value of *s*(*t*) when the action was selected, and by *s*′ we mean the value of *s*(*t*) when the action terminates (or the current time, if it has not yet terminated). We use the notation *s* and *s*′ for the sake of simplicity, and to connect to the standard RL notation.

The system needs to find appropriate output weights such that, given an input of *s*, the neurons will output *Q*(*s*). Since we do not know the correct *Q* function ahead of time, we cannot analytically determine the weights—they need to be learned, via the learning rule in Eq (5) (i.e., Δ*ω*_*ij*_ = *κα*_*j*_ *e*_*j*_ *Ea*_*i*_(*x*)). *E* in this case is an *n*-dimensional vector containing the error for each dimension (i.e., each action). In theory this could be a different error for each dimension, but in this model the error is equal to the TD error for the selected action and zero for all other actions. We will discuss how this error is computed in Section 4.4.

Examining the learning rule (Eq (5)) reveals another important challenge for learning action values: the weight update is based on the presynaptic neuron’s current activity, *a*(*x*). In the context of the action values component, the input is the state, so the update is dependent on the activity in the current state, *a*(*s*). The problem is that the TD error (Eq (2)) cannot be calculated until the agent arrives in *s*′, because it requires the comparison *Q*(*s*′, *a*′) − *Q*(*s*, *a*). At that point the input is *s*′, not *s*, so the neuron activity represents *a*(*s*′) rather than *a*(*s*). If the TD error is applied at that point, the effect would be to adjust *Q*(*s*′), not *Q*(*s*). Thus the model needs to somehow apply the learning update based on prior neural activities. This is not a problem purely computational approaches worry about, as they can simply store the previous activities and recall them when needed. However, a biological model needs to explain how this can be accomplished by a system operating in real time.

The problem is how to preserve the neural activity from when action *a* was selected, *a*(*s*), until *a* terminates. The standard approach to preserving neural activity over time is to use eligibility traces (e.g., [6]). For example, if we changed the learning update to be

$$\begin{array}{c} \Delta \omega_{ij}=\kappa \alpha_{j}e_{j}E\lambda \left(a_{i}\left(x\right)\right) \end{array}$$ (7)

where *λ* denotes a decaying eligibility trace, then the learning update would be applied based on the previous activity contained in the eligibility trace, which could be *a*(*s*). However, as discussed previously, eligibility traces can only preserve information over fixed time periods, and do not allow us to distinguish between actions with different hierarchical structure. In an SMDP/HRL environment there is an unknown and potentially lengthy period of active processing separating *s* and *s*′, so there is no guarantee that *λ*(*a*(*x*)) will contain any useful trace of *a*(*s*). Thus some other method is needed.

**Fig 2:**
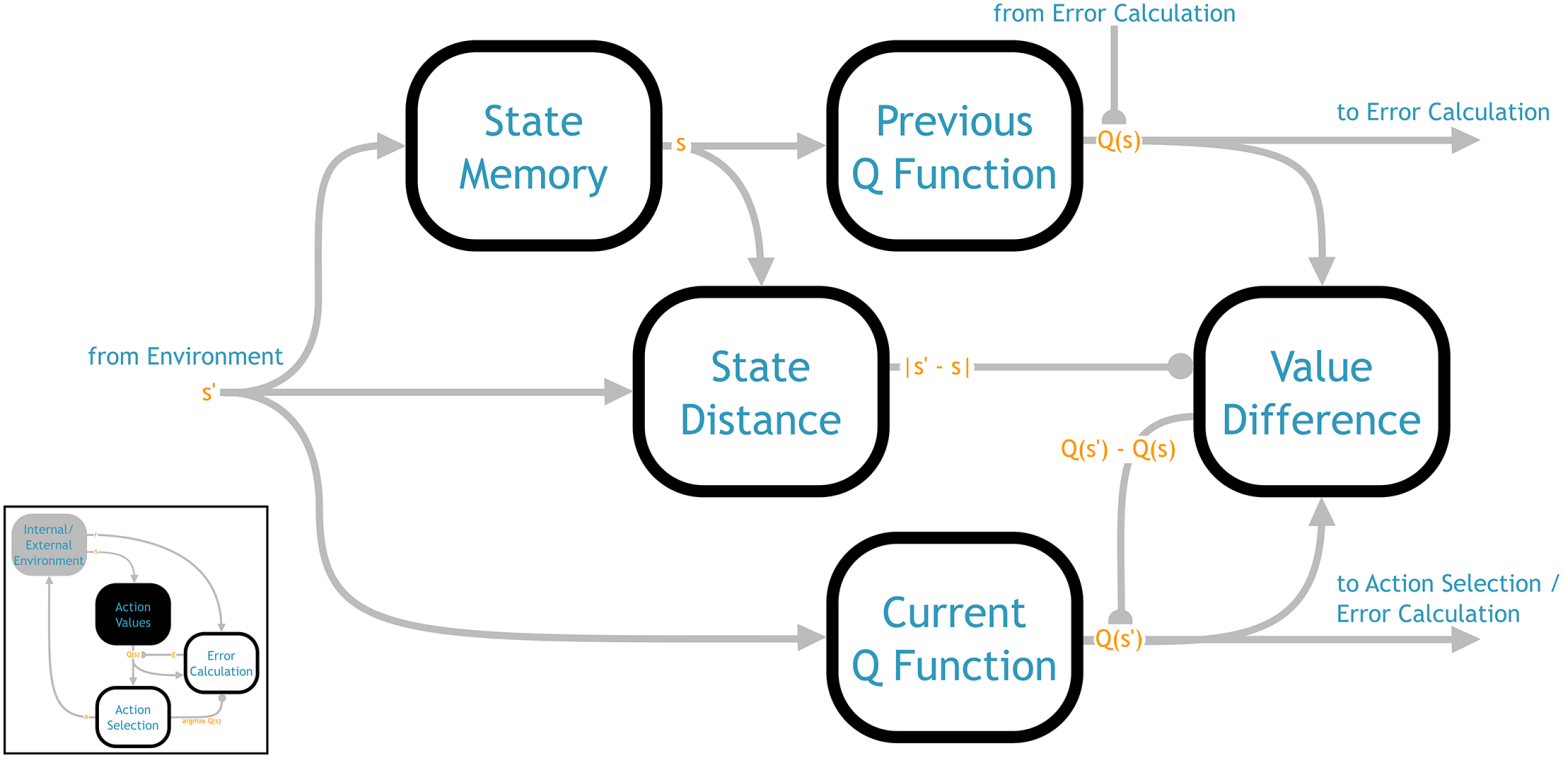
Architecture of the action values component (from Fig 1). This component computes the current and previous *Q* values (*Q*(*s*′) and *Q*(*s*)) based on the current and stored state, respectively. The previous *Q* function is trained by the TD error signal from the error calculation component. The current *Q* function is trained to match the previous *Q* function output whenever the distance between the current and previous state is below a threshold. The output of *Q*(*s*) and *Q*(*s*′) is sent to the error calculation component, and *Q*(*s*′) is sent to the action selection component.

In this model we solve the problem via a novel dual training system, shown in Fig 2. Rather than a single population representing the *Q* function, the network contains two populations. Both are representing the same *Q* function, but one receives the current state as input and the other receives the previous state (stored in a recurrently connected memory population implementing a line attractor, see [46]).

The TD update is only applied to the output weights of the previous state population. When the TD update is calculated in *s*′ the input to this population is still *s*, thus the appropriate *Q* values are updated, *Q*(*s*). However, action selection and error calculation need to be based on the *Q* values of the current state, *Q*(*s*′); this is why we require the second neural population that operates on the current state. The question then is how to update the output weights of the latter population.

The key idea is that the output of the previous state population can be used to train the current state population. Whenever the vectors representing *s* and *s*′ are the same (or within some distance in the continuous case), the output of the two populations, *Q*(*s*) and *Q*(*s*′), should be the same. Therefore the difference *Q*(*s*) − *Q*(*s*′) can be used as the error signal for *Q*(*s*′). That is,

$$\begin{array}{c} E=\{\begin{array}{cc} Q\left(s\right)-Q\left(s^{\prime }\right) & \text{if}\;∥s-s^{\prime }∥<\theta \\ 0 & \text{otherwise} \end{array} \end{array}$$ (8)

where *θ* is some threshold value. This error signal is then used to update the decoders via the same learning rule (Eq (5)). In this case the neuron activity is *a*(*s*′) and the goal is to update *Q*(*s*′), so there is no problem.

Thus all the connections shown in Fig 2 can be learned via the local learning rule of Eq (5), or represent fixed transformations where the standard NEF techniques can be used to find the weights. As mentioned, specific details on the latter can be found in [45].

### 4.3 Action selection component

**Fig 3:**
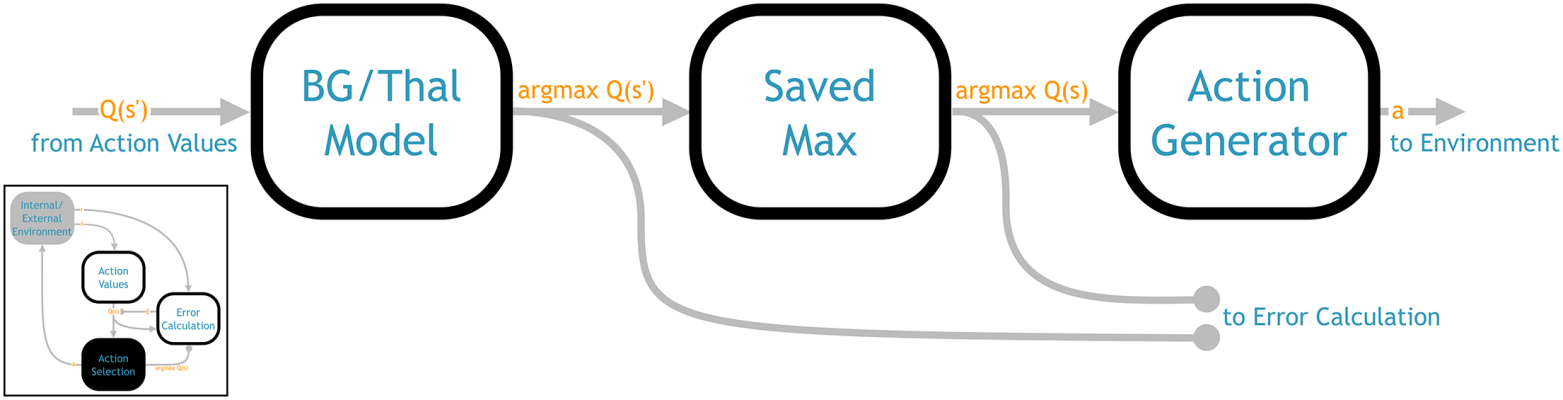
Architecture of the action selection network (from Fig 1). The leftmost component represents the model of the basal ganglia and thalamus described in [48]. The centre component stores the output of the previous component across the SMDP time delay. The rightmost component transforms the argmax vector into the vector associated with the selected action.

The task of the action selection component (Fig 3) is to select an action based on the output of the action values component. In other words, it needs to convert the *Q* values into a policy. The core of this component is a basal ganglia/thalamus model based on work in [47] and extended and translated into a detailed neural model using the NEF in [48]. The function computed by this network is an approximate arg max; given a vector of *Q* values as input, it will compute

$$\begin{array}{c} BG\left(s\right)=\overset{\left|A\right|}{\underset{i=1}{⨁}}\{\begin{array}{cc} 1 & \text{if}\;a_{i}=\underset{a}{\text{arg max}}Q\left(s,a\right)\\ 0 & \text{otherwise} \end{array} \end{array}$$ (9)

That is, a vector of the same length as *Q*(*s*), with 1 for the highest valued action and 0 elsewhere. Thus the basal ganglia model is well suited to computing a simple greedy policy, the NHRL model just needs to map the output of Eq (9) to the action vectors defined by the environment.

Exploration is accomplished in this model by adding random noise (drawn from $N(0,\sigma^{2})$) to the *Q* values coming from the action values component. The effect of this is akin to a soft-max policy, a common approach to exploration in RL. The probability that an action is selected is equal to the probability that the noise pushes the value of that action higher than the maximum action value. That is,

$$\begin{array}{ccc} \pi (s,a) & = & p(N\left(0,2\sigma^{2}\right)>\max Q\left(s\right)-Q\left(s,a\right))\\ & = & \frac{1}{2}-\frac{1}{\sqrt{\pi }}\int_{0}^{z}e^{-x^{2}}dx \end{array}$$ (10)

where $z=\frac{\max Q(s)-Q(s,a)}{2\sqrt{2}\sigma }$. Note that this expression has been slightly simplified, in that it only compares the given action to the maximum value action, and not all other actions. The action probabilities also need to be normalized by dividing by ∑_*a*_ *π*(*s*, *a*). The important intuition from Eq (10) is that the probability of selecting an action is proportional to how close that action is to the max, which is the essential function of the soft-max policy. This addition allows this component to implement all the required functions of an RL policy.

### 4.4 Error calculation component

**Fig 4:**
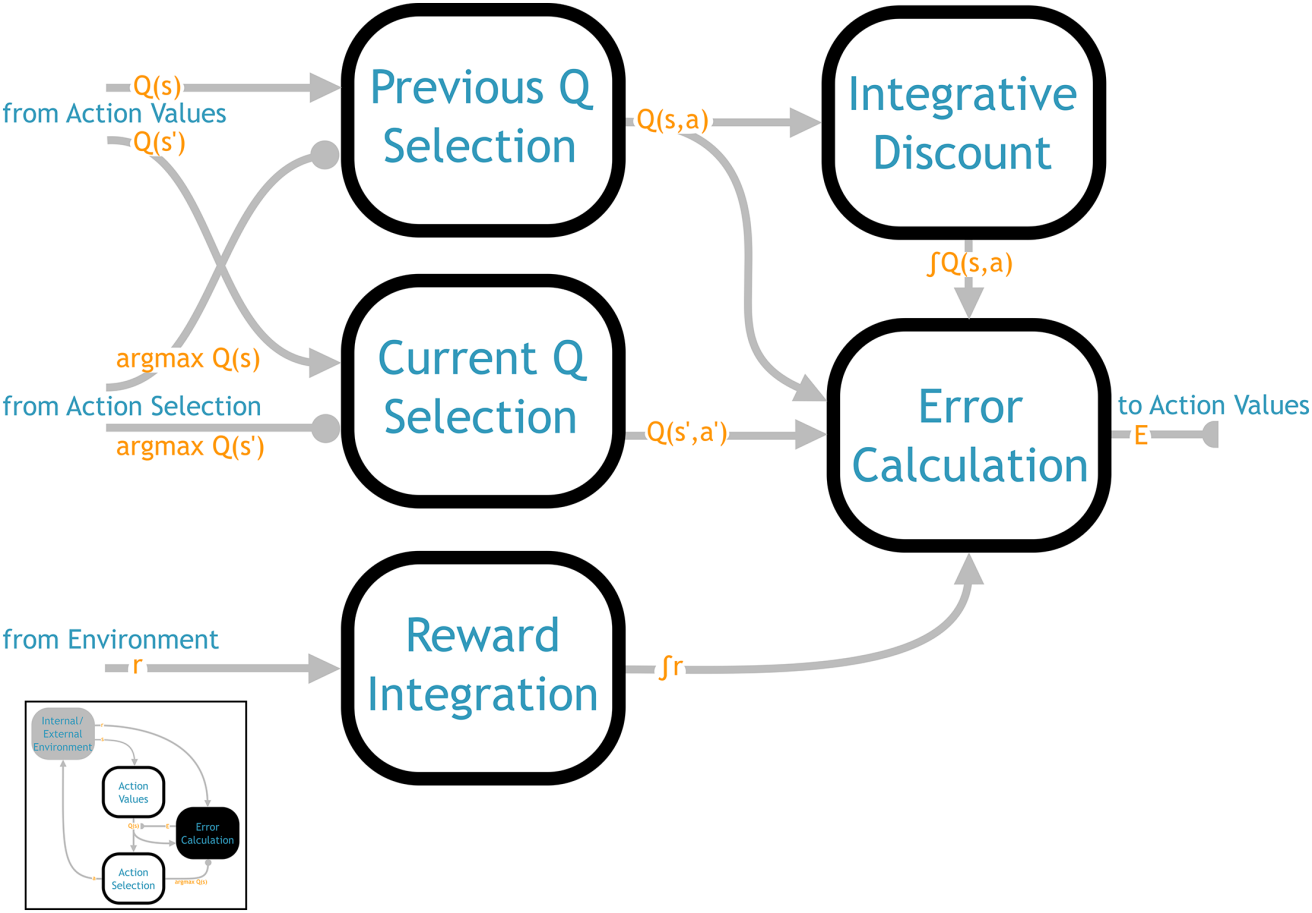
Architecture of the error calculation network (from Fig 1). Implements the SMDP TD error calculation of Eq (12). The final output is used as the error signal modifying the *Q* function representation in the action values component.

The purpose of the error calculation component (Fig 4) is to calculate the SMDP TD prediction error (see Eq (3)):

$$\begin{array}{c} \delta \left(s,a\right)=\sum_{t=0}^{\tau -1}\gamma^{t}r_{t}+\gamma^{\tau }Q\left(s^{\prime },a^{\prime }\right)-Q\left(s,a\right) \end{array}$$ (11)

There are four basic elements that go into this computation: the values of the current and previously selected action, the discount, and the reward.

The action values for the previous and current state, *Q*(*s*) and *Q*(*s*′), are already computed in the action values component, as described previously. Thus they are received here directly as inputs. The inhibitory input from the action selection component is used to select out just the values for the selected actions, *Q*(*s*, *a*) and *Q*(*s*′, *a*′).

The next element of Eq (11) is the discount factor, *γ*. Expressed in continuous terms, *γ* is an exponentially decaying signal that is multiplied by incoming rewards across the SMDP delay period, as well as scaling the value of the next action *Q*(*s*′, *a*′) at the end of the delay period.

One approach is to represent the exponentially decaying signal via a recurrently connected population of neurons. The connection weights can be computed using the techniques of the NEF, with the specified transformation being a scale less than one. This will cause the represented value to decay over time, at a rate determined by the scale. This value can then be multiplied by the incoming rewards and current state value in order to implement Eq (11). However, this approach relies heavily on multiplication, which is difficult to perform accurately in neurons, particularly when the values being multiplied are of very different magnitude (as they are likely to be here, depending on the range of the *Q* values). This inaccuracy can be problematic, because the TD error calculation often involves very slight differences in value that we do not want to disappear in the multiplicative noise. This motivated a second approach that would be less dependent on multiplication.

In the second approach to discounting we calculate the discount by integrating the value of the previous action. The advantage of this approach is that this discount factor can simply be subtracted from the TD error, rather than combined multiplicatively:

$$\begin{array}{c} \delta \left(s,a\right)=\sum_{t=0}^{\tau -1}r_{t}+Q\left(s^{\prime },a^{\prime }\right)-Q\left(s,a\right)-\tau \gamma Q\left(s,a\right) \end{array}$$ (12)

Note that rather than multiplying by *τ*, in practice *τγQ*(*s*, *a*) can be computed by integrating the *γQ*(*s*, *a*) signal across the delay period. Also note that while we describe these equations in terms of discrete time for continuity with previous equations, in the model all of these variables are continuous signals.

Clearly this form of discounting is not mathematically equivalent to the multiplicative discount. However, it captures the two basic properties of the multiplicative discount—namely, that the discount (*τγQ*(*s*, *a*)) scales with the length of the delay period and with the magnitude of the *Q* values. In addition, it accomplishes the practical purpose of the discount of keeping the expected value of the *Q* values from going to infinity (it can be thought of as a regularization term based on the magnitude of the *Q* values). This approach also bears many similarities to “average reward” TD formulations (e.g., [49]), which do away with the multiplicative discount but replace it with an average reward term. Tracking the average reward is another difficult neural operation, which is why we did not adopt that approach in this model, but it is an interesting avenue for future exploration. In the supplementary material (S1 File) we discuss the theoretical convergence properties of our modified error formulation.

In terms of implementing this discount neurally, there are well established techniques in the NEF for implementing a neural integrator using a recurrently connected population of neurons [29, 46]. Connecting the output of the population representing *Q*(*s*, *a*) to the integrator population with a scale of *γ* will result in the desired computation.

With *Q*(*s*, *a*), *Q*(*s*′, *a*′), and the discount all computed, the only remaining calculation in Eq (12) is to sum the reward. Again this can be accomplished using a recurrently connected population. The only difference is that in this case the input to the integrator is the reward signal.

Note that the output of this error calculation network will be a continuous signal across the delay period, whereas we only want to update *Q*(*s*, *a*) when the action *a* terminates. This can be achieved by inhibiting the output population, so that the error will be zero except when we want to apply the TD update. The timing of this inhibitory signal is based on the termination of the selected action, so that the learning update is applied whenever an action is completed.

### 4.5 Hierarchical composition of SMDP layers

This is the first neural model to implement the full SMDP cycle. As shown in Fig 1, this system takes a state from the environment, determines the values of the available actions in that state, picks an action based on the *Q* values, stores that information across the delay period, and when the action terminates it computes the SMDP TD error, updates the *Q* values accordingly, and repeats it all in the next state.

**Fig 5:**
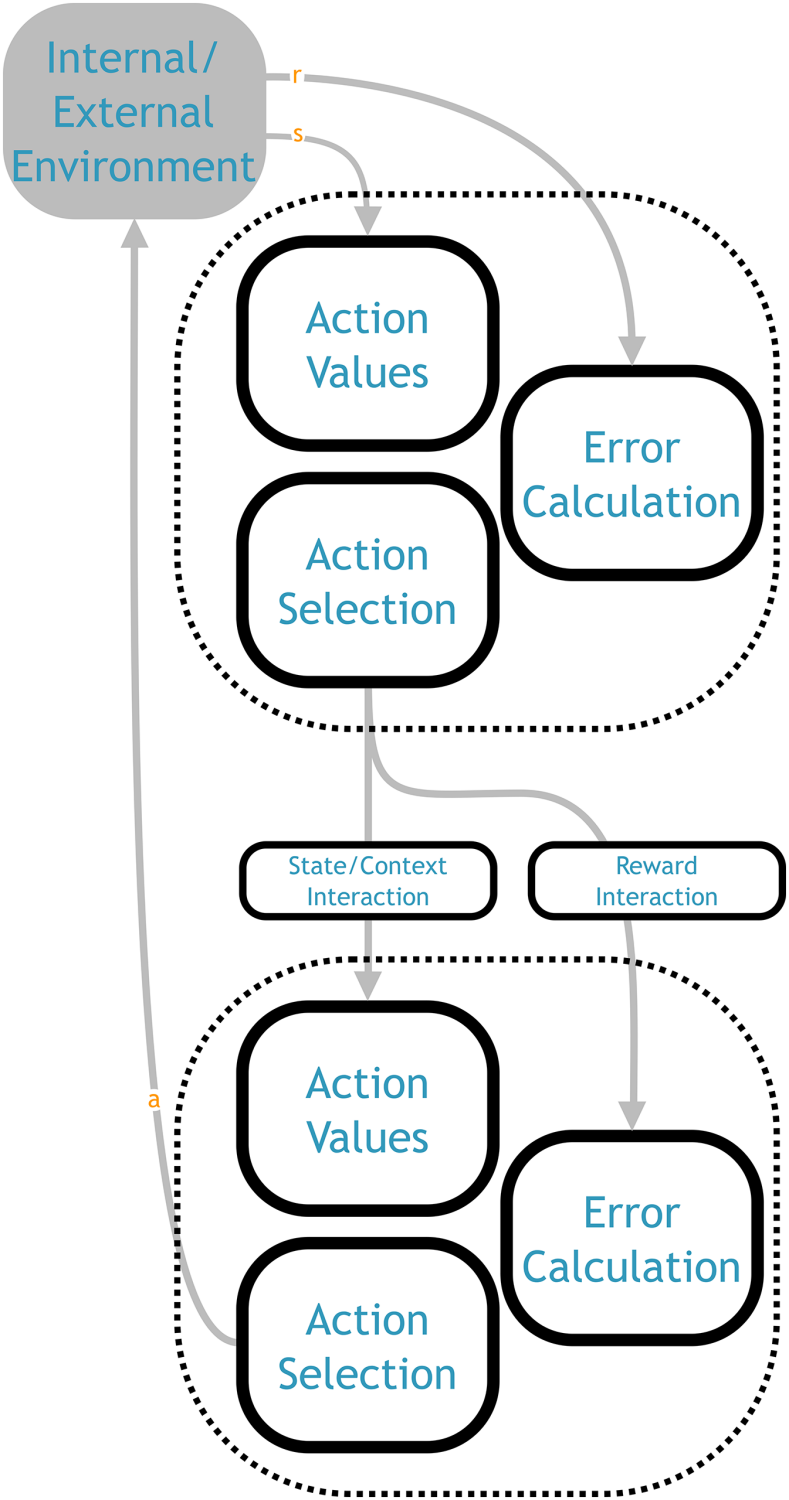
Hierarchical composition of the basic SMDP architecture (from Fig 1). The output of the higher level can modify either the state input to the action values component of the lower level (a state or context interaction), or the reward input to the error calculation component (reward interaction). Shown here with a two layer hierarchy, but this same approach can be repeated to any desired depth.

When extending the model to a hierarchical setting, we can think of this cycle as the operation of a single layer in the hierarchy (Fig 5). The only difference is that the delay until action termination represents the activity of a lower level, rather than a delay imposed by the environment. But from the perspective of the rest of the system, learning action values, selecting actions, and computing the TD error all proceed in the same way. Thus all that remains is to show how these basic elements can be composed into a multi-level hierarchical system.

In the design of this system we have tried to make the hierarchy as modular as possible. The goal is to be able to construct the hierarchy out of the basic pieces described above without modifying the internal operation of those pieces. This allows the model to smoothly scale to deeper hierarchies without an explosion of complexity. In practice what this modularity means is that the only interaction between layers is via the normal inputs and outputs of the RL process. That is, the layers interact via the same inputs and outputs with which the flat system interacts with the environment (state and reward inputs, and action output)—there are no new connections between the internal components of different layers.

In other words, from the perspective of a single layer, the layers above and below it are just a part of the environmental black box. The layer sends out some action vector, which through some unknown mechanism (the operation of other layers) results in some rewards being received as input and a new state when the action terminates. Interestingly, this bears many similarities to one of the earliest computational theories of hierarchical RL, the Feudal RL of [50]. The individual layers have no “knowledge” that they are embedded in a hierarchy; each of them is just working independently on their own SMDP reinforcement learning problem, trying to pick whichever actions will maximize their reward. Only from an outside perspective can it be seen that the RL problems of the different layers are connected to one another.

However, that still leaves open a wide range of possibilities as to what those inputs and outputs look like, and this model does not prescribe a specific implementation. As discussed previously, the goal here is to describe a generic model that can be applied to many different tasks. Different tasks will require different types of hierarchical interaction, depending on the hierarchical structure of the task. In the results section we will give examples of specific tasks and discuss how the interactions were implemented in those cases. Here we will speak more generally about the different styles of interaction and the functionality they afford. We will divide the ways in which layers can interact into three different categories: “context”, “state”, and “reward” interactions.

#### 4.5.1 Context interactions

In a context interaction the higher layer adds some new state information to the input of the lower layer. It is termed a context interaction because this extra state can be thought of as a context signal that modifies how the lower level responds to the normal environmental state. This concatenation can be implemented by a single population that receives both the environmental state and the output of the higher level as input, concatenates them, and then connects its output to the state input of the lower layer. The lower level then has as its input both the normal state *s* and an indication of the goal, or context, *c* (this is a common approach to context-based learning, e.g. [51, 52]). Recall that the lower level is not aware that it is part of a hierarchy, and so is unaware that its input is a composition of these two elements. It just sees some state input $\tilde{s}$, and it tries to learn the values of its available actions in each state.

The important feature that the context interaction allows is that the same environmental state *s* is now represented at multiple different points in the concatenated state space of *S* × *C*. This means that the agent can learn different action values for the same environmental state, depending on the context. For example, the house navigation robot could represent one set of action values if the high level context is “go to the kitchen” and a different set of action values for the context “go to the bedroom”.

#### 4.5.2 State interactions

In state interactions the higher level modifies the environmental state for the lower level, rather than appending new information. The primary use case for this is state abstraction, where aspects of the state irrelevant to the current subtask are ignored. This can be implemented via an inhibitory circuit that selectively gates the state vector based on the output of a higher layer. The output of that gating network then represents a new state $\tilde{s}$ that belongs to a subspace $\tilde{S}\subseteq S$, which becomes the state input for the lower layer. The lower level then only needs to learn a *Q* function over the smaller state space of $\tilde{S}$.

#### 4.5.3 Reward interactions

Reward interaction involves the higher level modifying the reward input of the lower level. The primary use case of this is to implement theories of pseudoreward—reward administered for completing a subtask, independent of the environmental reward [21, 22].

The pseudoreward can take on many forms, but generally speaking the low level should be rewarded for completing the goal set by the high level. It is difficult to specify the specific implementation of this mechanism in a task-independent fashion, as the notion of a goal will differ from task to task. However, the most common type of goal is likely to involve reaching some target state *s*_0_. In this case the output *a* of the high level defines the target state *s*_0_ (i.e., there is some one-to-one mapping *A* ↦ *S*). The pseudoreward signal can then be computed via, e.g.,

$$\begin{array}{c} r_{t}=\{\begin{array}{cc} 1 & \text{if}\;∥s-s_{0}∥<\theta \\ 0 & \text{otherwise} \end{array} \end{array}$$ (13)

Note that this function has the same form as the error signal in the dual training system (Eq (8)). Thus the same circuit design used to compute that function can be reused here to compute the pseudoreward.

Although we have presented the different interaction types independently, in practice useful hierarchical structures often combine all three. For example, if state interaction is combined with context interaction, then the efficiency gains of the state abstraction can help balance the state space enlargement of the added context information. And it is often the chunking of the overall task into simpler subgoals, made possible by pseudoreward, that motivates the added context information, and allows for the simplifications of state abstraction.

The important feature of all three interaction types is that they operate only through the normal “flat” inputs and outputs. All that changes are the vector values of those signals, based on relatively simple transformations as described above. There are no changes made to, e.g., the internal action selection or TD error calculation of the components. This makes it easy to define different hierarchical interactions for different tasks, as it is not necessary to make changes to the fundamental implementation of the SMDP RL mechanisms.

### 4.6 Neuroanatomical mapping of the model

In the previous sections we discussed how the various model components can be implemented via the low-level features of the brain, namely neural activity and synaptic connections. Now we turn to how this model can be implemented via the macro-level structures of the brain. That is, where do the various components of this model lie physically in the brain?

**Fig 6:**
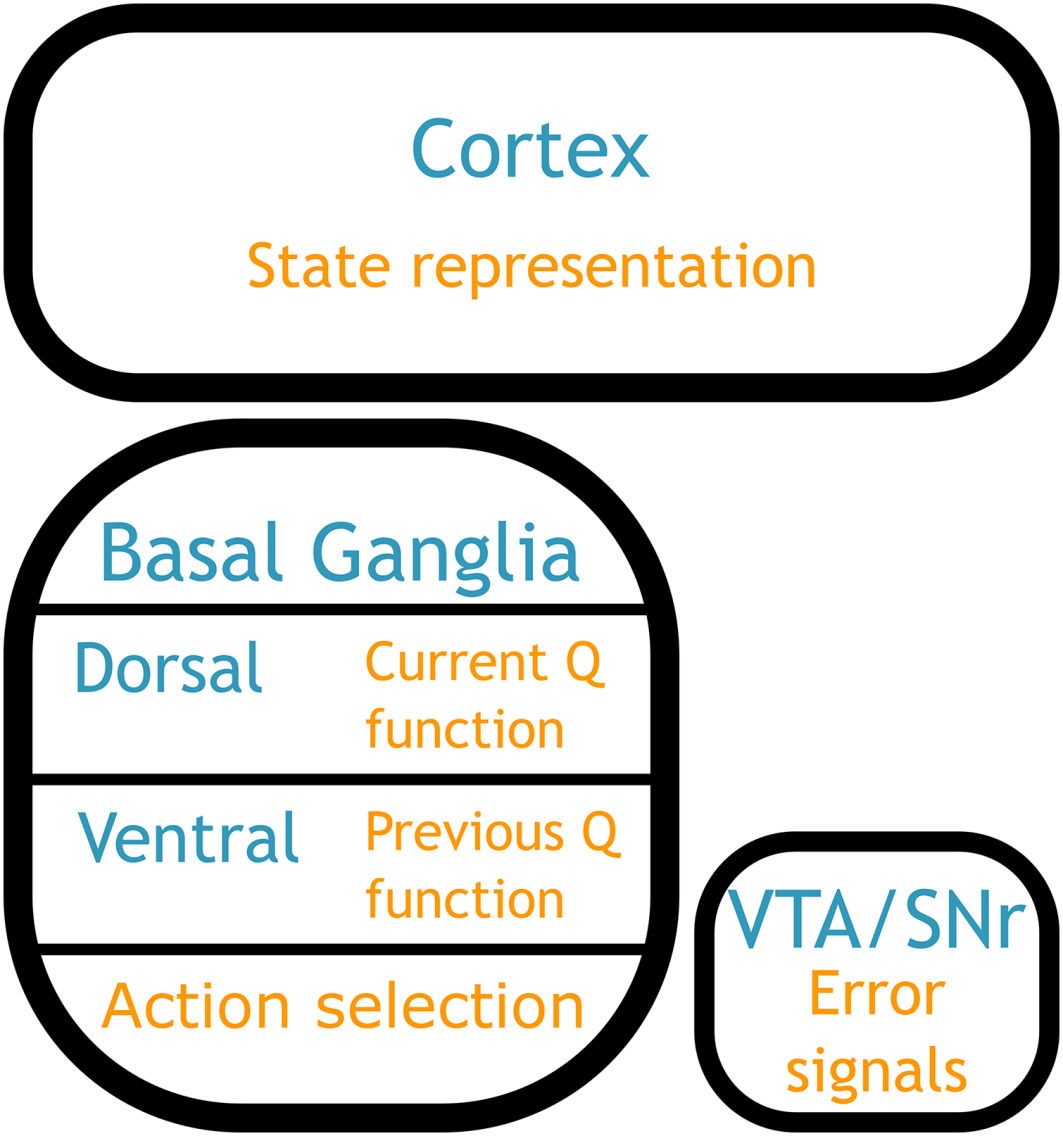
Neuroanatomical mapping of the model. **state representation**: input could be taken from almost any brain region, depending on the task. **action values**: *Q* values are represented in the striatum, divided into dorsal (**current Q function**) and ventral (**previous Q function**); this dorsal/ventral separation is a novel prediction of this model. **action selection**: the selection of a single output action based on *Q* values is performed in basal ganglia/thalamus. **error calculation**: the error signals used to update the *Q* functions are output from the ventral tegmental area/substantia nigra.

Extensive effort has gone into the search for neurophysiological correlates of RL processes (see [2, 40, 53] for reviews). In most cases the structure of this model is consistent with that past work, based on a cortico-striatal-thalamic action selection loop, with learning in the striatum modulated by dopaminergic inputs from midbrain nuclei (Fig 6; see [45] for more detail). Here we focus on the neuroanatomical mapping of the more unusual components of the model.

#### 4.6.1 Dual training system

One such feature is the dual training system in the action values component. This is a novel feature of this model, and so not something that has been directly investigated experimentally. The first issue to be addressed is where the previous state information is stored. Broadly speaking, we could think of any memory system as encoding previous state information. However, one area that seems to be particularly involved in memory for the purpose of reinforcement learning is the orbitofrontal cortex [40, 54]. For example, in [55] the authors found that neurons in the OFC consistently showed sustained activity following each state change in a delayed decision task. Importantly, this OFC activity was only, or predominantly, present when the monkey was in a rewarded, reinforcement learning environment. This suggests that this OFC activity is not just a generic memory system, but memory specifically tied to the reinforcement learning process, making it a possible candidate for the storage of previous state information in the dual training system.

The next key aspect of the dual training system is the internally generated error signal used to update the current *Q* function, based on the difference between the output of the two *Q* functions. This requires a particular form of connectivity, where the output of the two functions is combined and then fed back to just one of the functions. Interestingly, this is exactly the structure of the connectivity between ventral and dorsal striatum via the dopaminergic nuclei, reviewed in [56]. The interesting observation highlighted in [56] is that there is an asymmetry in the connections between the dorsal/ventral striatum and the dopaminergic nuclei. The ventral striatum projects broadly throughout the dopaminergic nuclei, while the dorsal striatum only projects to a subset of the dopamine neurons. Both receive projections back from the dopamine neurons, but the result of this connectivity pattern is that only the dopamine neurons projecting to the dorsal striatum receive input from both ventral and dorsal striatum. In other words, those neurons could compute the difference between the dorsal and ventral output, and project that difference back to the dorsal striatum. Another important observation is that the OFC projects to ventral, but not dorsal, striatum. Thus the hypothesized previous state information would be available to the ventral striatum.

This separation of the previous and current *Q* functions between ventral and dorsal striatum is a novel prediction of this model. It is also important to emphasize that this neuroanatomical mapping is speculative, as the novelty of the dual training mechanism restricts us to indirect evidence. Although the mapping we have established here is plausible, it is entirely possible that new data could invalidate this proposal, or create a more convincing mapping elsewhere. In the discussion we will present some of the specific predictions that arise from the dual training system, and how they could be used to experimentally investigate the mapping we propose here.

#### 4.6.2 Hierarchical neuroanatomical structure

Another unique feature of the NHRL model is the extension to a hierarchical framework. Unfortunately, empirical data on hierarchical reinforcement learning is more difficult to come by than standard RL. One reason is that HRL is simply a younger field, and has had less time to accumulate data; the neural correlates of reinforcement learning have been under investigation since at least the mid-90s, whereas data on HRL has only started to appear within the last 5–7 years. Another challenge is that HRL tasks are required to be more complex, in order to provide a hierarchical structure. This makes them difficult to apply to animal models like rodents, or even primates, without simplifying the hierarchies to the point of triviality. Thus much of the HRL data come from human studies, using non-invasive methods such as fMRI.

This model suggests that the different processing levels are represented by physically distinct neural networks. This is consistent with the observation of multiple, parallel cortico-striatal-thalamic loops [57]. In addition, more recent results have shown that these loops are actually more of a spiral, with information from “higher level” cortical areas spiralling through the basal ganglia down into “lower level” areas [58]. Finally, we can place these multiple levels in a roughly caudal–rostral gradient, with higher levels of the hierarchy occupying increasingly anterior regions of striatum/cortex [59].

However, it is important to note that although the simple repetition of this mapping is appealing, as we move to increasingly high level RL processing there may be qualitative differences in the neural circuitry. For example, although we have proposed that the basic representation of action values occurs in the striatum, there is evidence that prefrontal regions such as anterior cingulate cortex or OFC may be involved in representing action values [40, 55, 60–62]. These signals are still relayed through the striatum, thus we would still expect to see striatal activity correlated with action values. The difference is in where those values are being computed. The resulting neuroanatomical mapping will depend on the task-specific implementation; here we only want to highlight the fact that such an extension is compatible with the rest of the mapping presented here.

It is also important to note that hierarchical processing need not be implemented by hierarchical neuroanatomical structure, although that is the approach we have taken in this model [63, 64]. In the supplementary material (S1 File) we discuss how the architecture we present here could be adapted so as to implement hierarchical processing via recurrent dynamics.

## 5 Results

The goals of this section are threefold. First and foremost, the purpose of these results is to demonstrate that the model works—that the action values, action selection, and error calculation components all perform the functions described above, and that together they are able to carry out the hierarchical reinforcement learning process. The second goal is to demonstrate that the model’s performance is consistent with neurophysiological data, in order to further support its biological plausibility. And third, we seek to demonstrate the advantages of hierarchical learning, in order to show the benefit of including these features in models of decision making.

Here we will focus on the overall function of the model. See the supplementary material (S1 File) for demonstrations of the operation of individual components within the model. In each section we will begin by discussing how the model is applied to that particular task (which we call the “task implementation”). This involves making specific decisions about the general model architecture described in the previous section (e.g., specifying the context/state/reward interactions between layers). We make no task-specific modifications to the model other than those described in each section. We then present results from that task, and discuss the neural/computational implications of those results.

### 5.1 Variable delay navigation task

As discussed previously, in terms of basic (non-hierarchical) RL the model in [12] is closest in function to the one we present here. Here we compare the performance of the two models, in order to establish the baseline performance of the NHRL model (see [65] for more detail).

#### 5.1.1 Task implementation

**Fig 7:**
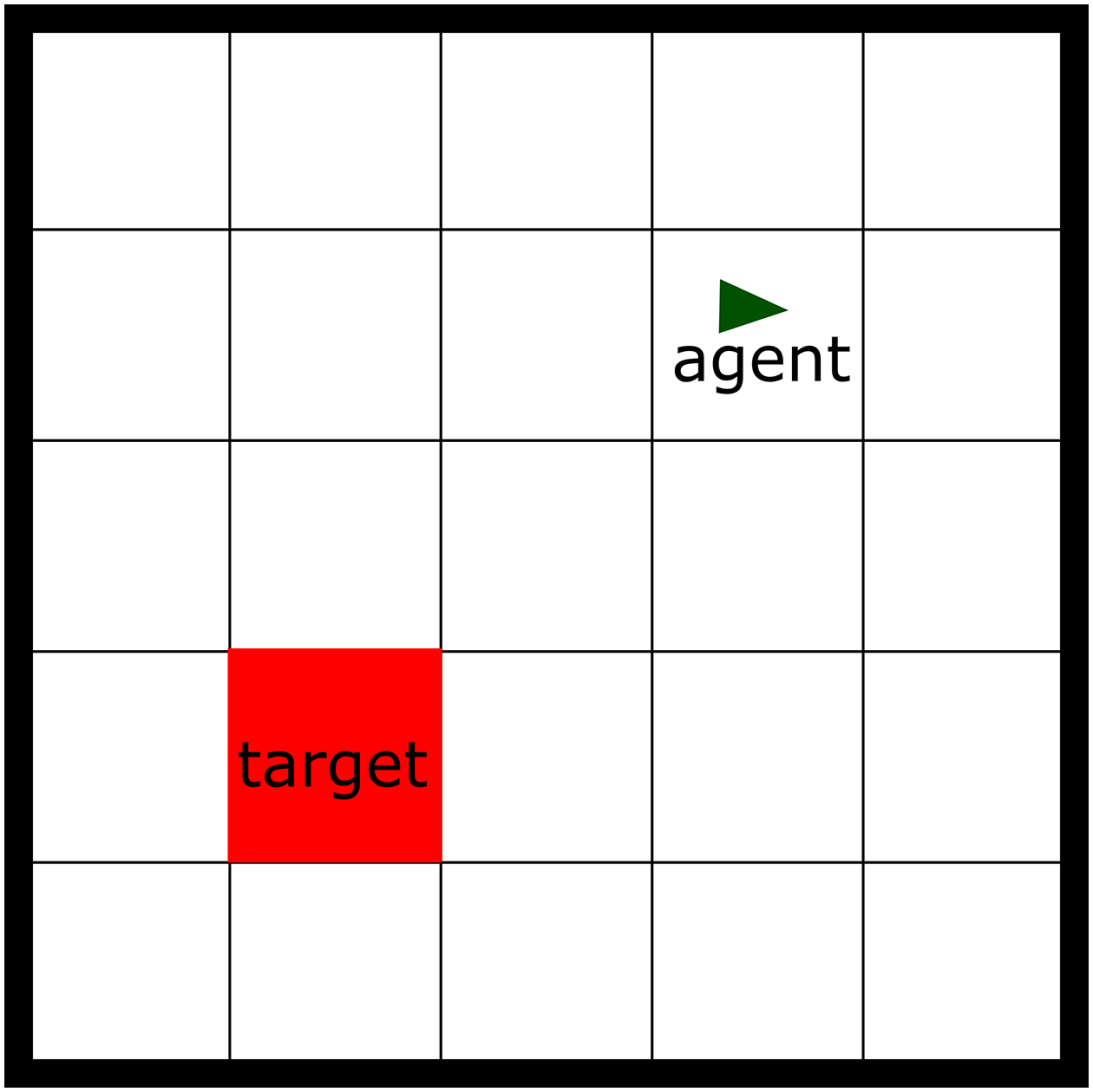
Variable delay navigation task. The environment is represented as a discrete 5x5 grid; the agent begins in a random location, and must navigate to the fixed target location.

The task used in [12] is a 5 × 5 grid navigation task (Fig 7). The agent begins in a random location, and must navigate to a fixed target location. The state is the *x*, *y* grid location of the agent. The NHRL model is designed to work in continuous space, so such an environment is somewhat unnatural. However, we approximate the discrete environment by moving the agent only to fixed points in the continuous space, representing the centres of the cells in the grid. The environment makes four actions available, each of which move the agent one square to the north, south, east, or west (unless the agent is at the edge of the grid in which case it stays in the same place). In the model in [12] state transitions occur instantaneously after the model makes a decision. In our model we use a random time delay of 600–900ms between state transitions, in order to demonstrate the ability of the model to perform in an SMDP environment. The agent receives a reward of 1 in the goal state, and 0 at all other times.

#### 5.1.2 Performance

**Fig 8:**
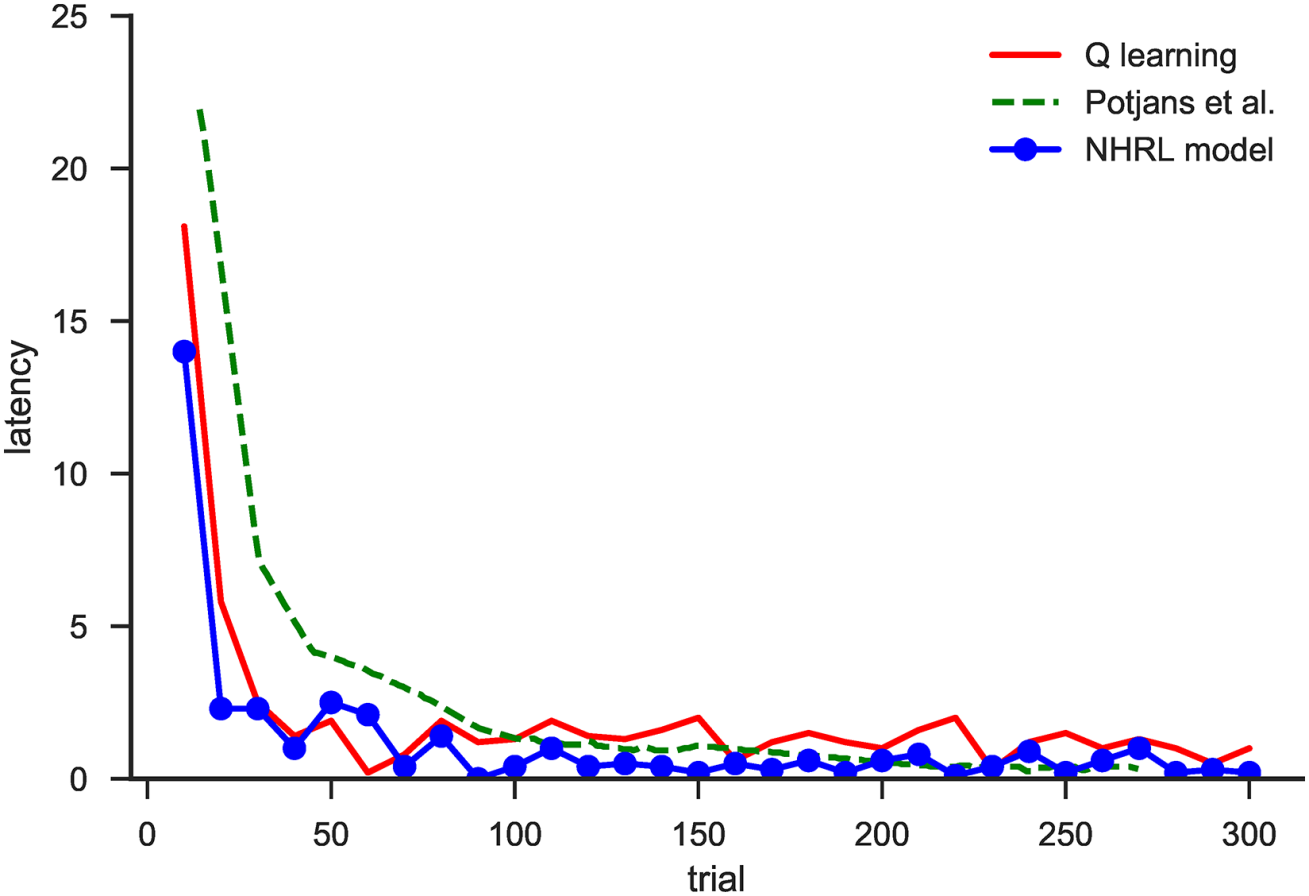
Performance of the model on the discrete variable delay navigation task. Latency describes the number of steps taken by the model to reach the goal in excess of the optimal number of steps. Performance is shown in comparison to a simple algorithmic Q-learning implementation and the model from [12].

Fig 8 shows the performance of the model on this task. A trial begins when the agent starts in a random grid cell, and ends when it reaches the target. The “latency” measure refers to the difference between how many steps the agent took to reach the target and the optimal number of steps (the Manhattan distance from the start location to the target). The “Q learning” line shows the performance of a simple table-based Q-learning implementation, for comparison.

It can be seen that all three implementations are able to learn the task. The model from [12] is somewhat slower to learn, but within 100 trials all have achieved near-optimal performance. It is also important to note that the model we present here is operating on a variable delay version of the task, which could not be solved by the model from [12]. However, the main conclusion to draw from this result is simply that the model’s basic RL performance is at least as good as the most similar neural model. We now expand that performance into the domain of HRL.

### 5.2 Delivery task

#### 5.2.1 Task implementation

**Fig 9:**
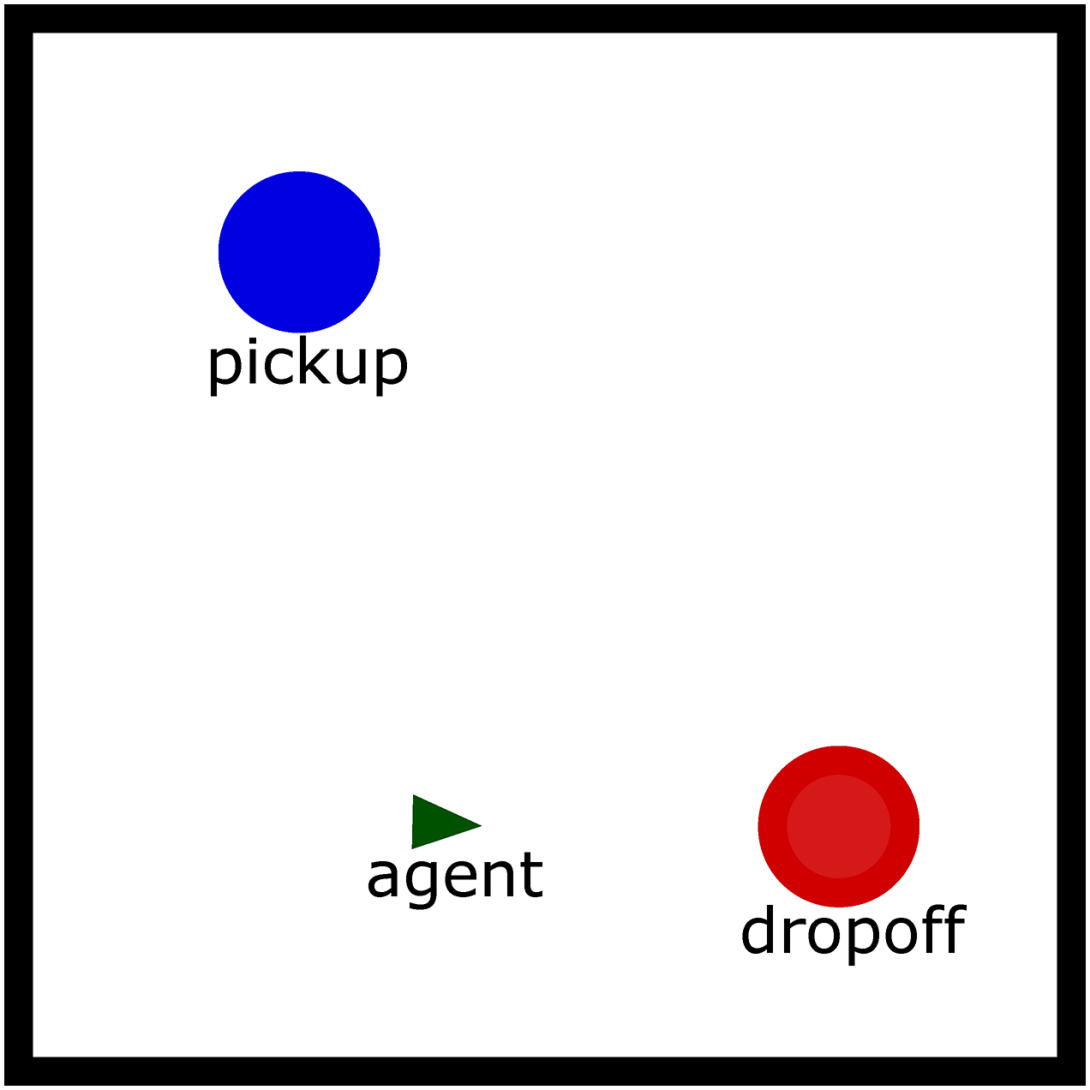
Delivery task. The agent begins in a random location, then must navigate to the pickup location to retrieve the package followed by the delivery location to receive reward. Unlike the variable delay task, here the location is represented as a continuous variable.

Next we explore the performance of the model on a common hierarchical task, referred to as the delivery task (Fig 9) [22]. The agent must move to a pickup location to retrieve a package, and then navigate to a dropoff location to receive reward. As in the previous task, the environment provides four actions to the agent, corresponding to movement in the four cardinal directions. As before, the agent selects an action every 600–900ms. However, in this case the agent’s position is represented as a continuous point in 2D space, instead of discrete grid locations. Rather than the raw *x*, *y* position, in this task the environment represents the agent’s location using a simple approximation of place cell activations; “cells” are randomly distributed throughout the map, and each has a Gaussian activation based on the Euclidean distance from the agent to the centre of that state cell. The resulting vector of state cell activations is then concatenated with one of two vectors ([1, 0] or [0, 1]) indicating whether the agent has the package in hand or not, in order to form the final state representation (see [66] for more detail).

In this task we seek to demonstrate the advantages of a hierarchical system by comparing the performance of a hierarchical versus flat implementation. The hierarchical model has two layers. The lower layer has four actions, corresponding to the basic environmental actions (movement in the cardinal directions). The higher level has two actions, representing “go to the pick-up location” and “go to the delivery location”. The layers interact via a context interaction. The output of the high level (e.g., “go to the pick-up location”) is represented by a vector, which is appended to the state input of the lower level. Thus the low level has two contexts, a “pick-up” and “delivery” context. The high level can switch between the different contexts by changing its output action, thereby causing the agent to move to either the pick-up or delivery location via a single high level choice.

The low level receives a pseudoreward signal of 1.5 whenever the agent is in the location associated with the high level action (i.e. if the high level is outputting “pick-up” and the agent is in a pick-up state, the pseudoreward value is 1.5). At other times the pseudoreward is equal to a small negative penalty of -0.05. Thus when the high level outputs “pick-up” the low level will learn to maximize the pseudoreward in that context, which means learning a policy that will bring it to the pick-up location.

The flat model consists of just one layer with the four basic actions, similar to the lower level in the hierarchical model. However, this layer directly receives the environmental state and reward as input, without the context/reward interactions of the higher layer.

#### 5.2.2 Performance

**Fig 10:**
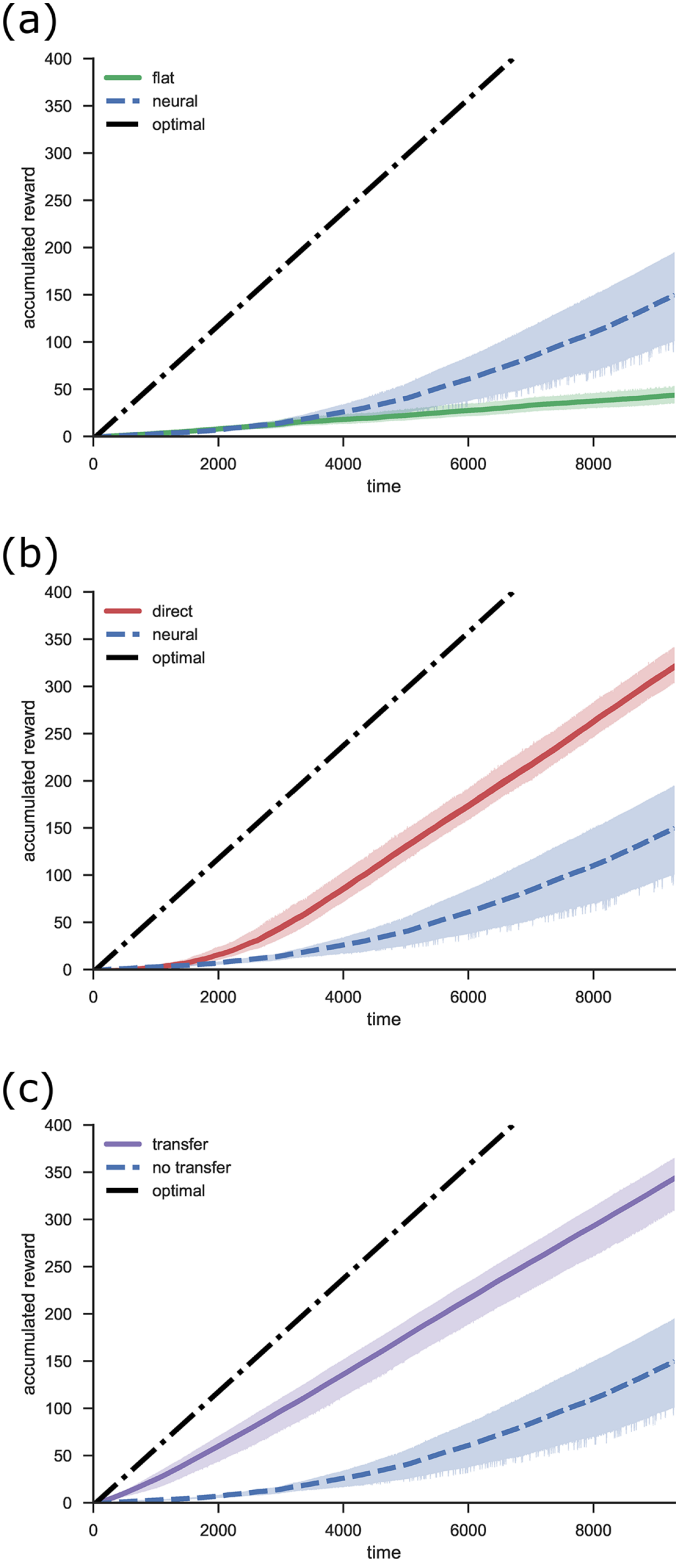
Performance of the NHRL model on the delivery task. Results are adjusted such that random performance corresponds to zero reward accumulation. The optimal line indicates the performance of an agent that always selects the action that takes it closest to the target. a) Performance of a flat versus hierarchical model on the continuous delivery task. b) Performance of the neural model versus a direct computational implementation (with perfect precision). c) Performance of a hierarchical model where low level skills were transferred from previous learning.

Fig 10a compares the performance of the model with and without hierarchical structure. The figure shows the total accumulated reward over time. Since this is the measure that the model seeks to maximize, the final point of this line indicates the agent’s overall performance. Another useful measure is the slope of the line, which represents the rate of reward accumulation. This indicates how well the agent is performing at any point in time, independent of previous performance. In all figures the time axis indicates simulated time (in seconds), that is, time as experienced by the agent. It takes the agent about 3 seconds to complete one iteration of the task if it moves optimally. The simulations in Fig 10a take 80–90 hours to run in real time, on a Dual Intel Xeon E5-2650 2GHz CPU.

We run the model five times in each condition, using different randomly generated parameters each time (this includes variables such as neuron encoders, gains, and biases, state cell locations, and exploration noise). The shaded area shows 95% confidence intervals for the mean.

It is clear that the hierarchical model’s overall performance is better than the flat model’s, in that it accumulates much more reward. Interestingly, we can also see that the hierarchical model’s instantaneous performance, as measured by the slope of reward accumulation, is higher at the end of the experiment (52% of optimal versus 9%), and the hierarchical performance is still improving whereas the flat model has a constant slope. In other words, it is not just that the hierarchical model learns more quickly, it also learns a better policy than the flat model. The reasons for this are not obvious. The state input to the lower level contains the same information in either case, and the same environmental actions are available, so it would seem that the flat model should be able to learn an equivalent policy.

The main reason for the improved performance of the hierarchical system lies in the reward propagation. Due to the discount factor, the shape of the action value function is a gradient decreasing outwards from the rewarded states. The discount goes to zero as the *Q* values approach zero, so the gradient will eventually flatten out. This means that the potential range of the *Q* values decreases the farther the agent moves from the target. This is significant because the action selection component has limited precision; if the *Q* values are too close together, then the basal ganglia model cannot tell them apart. We can observe this effect more directly if we change the action selection and error calculation components to a direct implementation of the underlying computations, rather than a neural approximation. The action values component is still implemented neurally (since we need the neurons to approximate the continuous *Q* function), but this means that the action selection and error calculations are performed with perfect precision. As we can see in Fig 10b, a model with perfect precision performs better than the neural model (reaching a peak performance of 73% of optimal versus 52%), despite the underlying mathematical computations being the same. This illustrates the special challenges facing a more biologically constrained neural model of reinforcement learning.

The hierarchical framework helps to reduce this problem, because HRL reduces the reward propagation distance. This occurs in two ways. First, the high level actions effectively represent shortcuts across the state space. When the high level selects the “delivery” action, from its perspective it reaches the target location in a single state change. Thus the effective distance of the previous state from the reward is much shorter, and the gradient is steeper. Second, the lower level benefits from the pseudoreward interaction. The environmental reward is only administered in the delivery location, thus in the flat model the reward has to propagate from the delivery location, back through the pick-up location, and then outwards from there. The pseudoreward, on the other hand, is associated directly with both the pick-up and delivery locations, depending on the action selected by the high level. Thus in any given state the lower layer in the hierarchical system has a shorter distance to travel before it reaches a rewarding state. These two factors combine to allow the hierarchical model to perform more successfully than the flat model.

One interesting aspect of this result is that this advantage only exists in biological (or, more generally, noisy/imprecise) systems. A purely computational system can always distinguish the *Q* values, no matter how small their range may be. Thus in these systems the flat and hierarchical models would always converge to the same performance in the long run, the only difference would be the learning speed. This demonstrates that when we incorporate HRL into neural models we do not simply recreate the computational advantages—we can find important practical benefits specific to the constraints faced by neural systems.

As discussed previously, another advantage of HRL is that the abstract actions represent modular, reusable subpolicies that can be transferred between tasks. We would like to demonstrate that this computational advantage is retained in the neural implementation. To do so we created a simpler version of the delivery task, where the agent is just rewarded for moving to a target location. There are several different locations where the target could be, and the environment indicates which target is active by appending a context vector to the state cell activations. Note that this environment effectively recreates the subgoal structure of the lower layer in the delivery task; there are several different locations that the agent needs to learn to move to, depending on some context vector appended to the state. The only difference is that those contexts are randomly picked by the environment, rather than governed by the hierarchical pick-up and delivery structure.

We then train a flat model in this environment. This model therefore learns the lower level policies needed in the delivery task—a set of *Q*(*s*⊕*c*, *a*) values that cause it to move to the target location associated with *c*—but not the high level structure of the delivery task. After 2000 seconds of training, we then add an upper layer as described above, and place the model in the delivery task.

Fig 10c shows the result of this knowledge transfer, resulting in a dramatic improvement in performance. It is important to note that this improvement goes beyond a simple 2000 second head start. The model with transfer very quickly reaches peak performance (66% of optimal), while the naive model is still learning at the end of the trial (52% of optimal). This highlights an important advantage of transfer learning, namely that of incremental training. The upper level’s learning problem is significantly complicated if the lower level does not reliably carry out its commands—from the upper level’s perspective, this will look like an SMDP with a highly stochastic transition function. This means that the initial exploration time of the upper level is largely wasted, because it is learning values based on an inaccurate lower level. In the transfer case the upper level is immediately presented with a reliable lower level, thereby easing its learning problem. In other words, learning multiple layers simultaneously is more difficult than learning one layer at a time, and knowledge transfer enables the latter approach.

### 5.3 Hierarchical stimuli task

The second hierarchical test for the model is a recreation of the task from [59]. In this task subjects are shown a stimulus and must press one of three buttons. After pressing the button they receive feedback on whether or not they pressed the correct button. The stimuli are artificial shapes that vary in colour, shape, and orientation. There are two colours, three shapes, and three orientations, for a total of 18 stimuli, and therefore 18 stimuli–response mappings that need to be learned.

**Fig 11:**
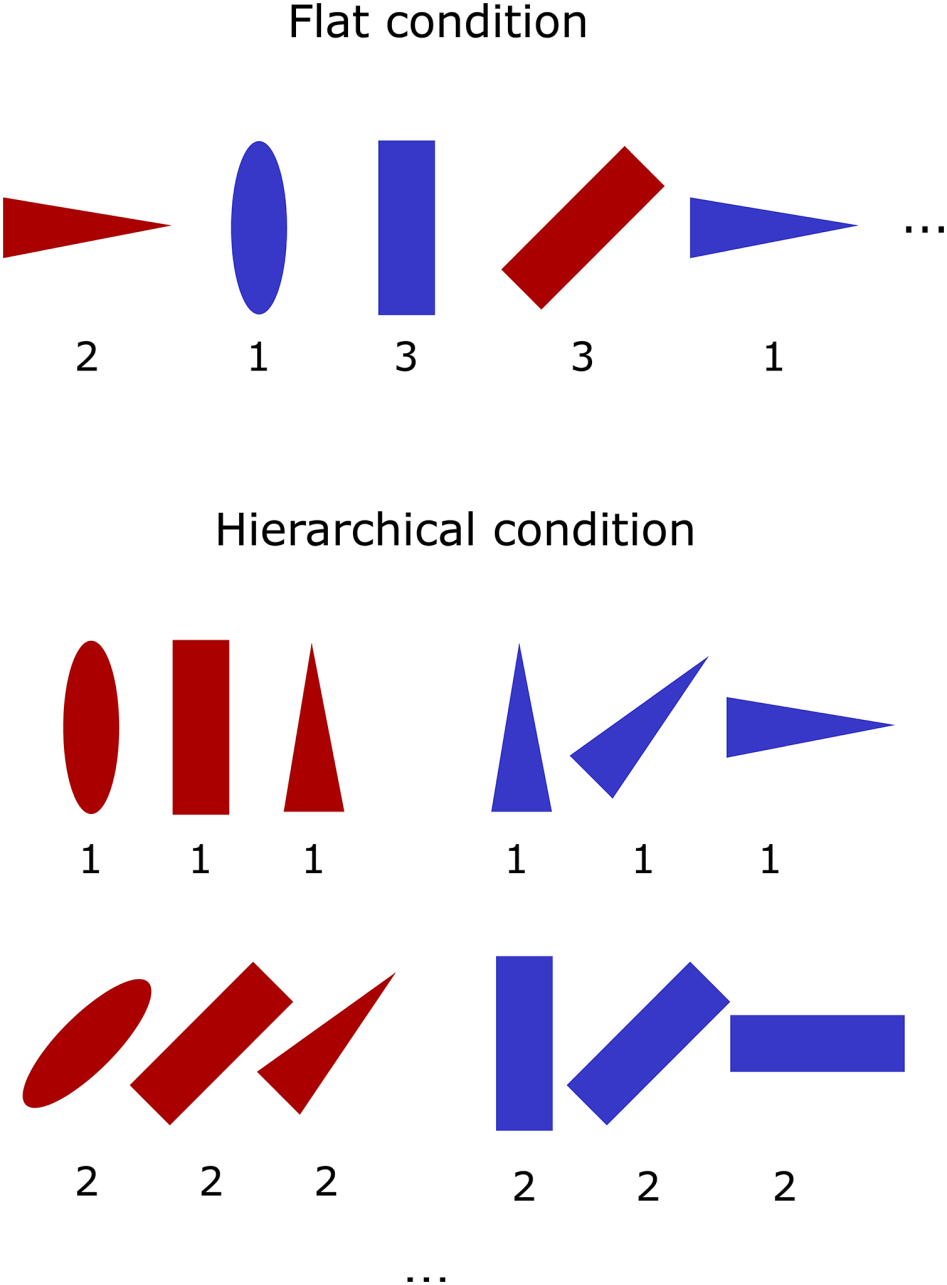
Hierarchical stimuli task. In the flat condition, random responses (1–3) are assigned to each shape/colour/orientation combination. In the hierarchical condition, responses are organized according to colour. For red objects the response is dependent on the orientation (regardless of shape), and for blue objects the response is dependent on the shape (regardless of orientation).

In order to investigate hierarchical processing, the authors created two versions of the task (Fig 11). In the “flat” version each stimulus was mapped to an arbitrary button—there were no patterns in play, the subject just had to separately learn the correct button for each stimulus. In the “hierarchical” version of the task the button presses follow a rule. If the object has one colour, the button press is dependent on the shape (i.e., button A for shape 1, button B for shape 2, etc.) regardless of the orientation, and vice versa if the object has the other colour. The idea is that the difference in processing between the two tasks will reveal the effect of the brain’s hierarchical processing components.

From a computational perspective, this task is not as interesting as the previous one. Each button press is independent, so there are no temporal sequences of actions involved; this is an associative RL task. The reason we chose this task is that it provides unique neural results that can be used to test potential HRL implementations, as we will see below. This is important, because we do not just want to show that the NHRL model can perform HRL abstractly, we want to show that the specific implementation of HRL we have developed in this model is a plausible hypothesis for how the brain could perform HRL. In addition, the closest hierarchical model to the one we present here was applied to this task [18]. Thus we can examine how two different hierarchical systems model data on the same task.

#### 5.3.1 Task implementation

In the previous two tasks we used the firing rate formulation for the LIF neurons that make up the model. For this task, since the goal is to compare as closely as possible to neural data, we use the spiking LIF model. We make no other changes to the structure of the model, simply swap out the neuron model (this fluidity is made possible by the Nengo simulation environment [34]). Thus these results also demonstrate that rate and spiking neurons can be used interchangeably in this model.

The state in this task is the stimulus object. We follow the implementation of [18] in representing the stimulus via an 8-dimensional vector. The vector has one element for each possible value of the stimulus; the two colours are represented by the two vectors [1, 0] and [0, 1], the three shapes are [1, 0, 0], [0, 1, 0], and [0, 0, 1], and so on. In the model in [18] each attribute is represented as a separate input, but in our model we concatenate the three attribute vectors together to form the full stimulus representation (e.g. [1, 0, 1, 0, 0, 1, 0, 0]), which becomes the state input to the model.

Each stimulus is presented for 500ms. During this time the model selects one of the three output actions (corresponding to the three buttons). After 500ms the environment checks the action to see if it is correct or not, and delivers a corresponding reward of ±1.5 for 100ms. It then randomly picks a new stimulus and the process repeats.

In this task the high level has two actions, corresponding to the two rules “respond according to shape” and “respond according to orientation”, with a third option of producing no output, indicating no rule. The lower level has the three basic actions representing the three button presses.

In this task we make use of state abstraction. When the high level selects the “shape” rule, what this means is that all the state information other than shape is irrelevant. In other words, the 8-dimensional state can be projected on to the 3-dimensional shape space. In the model we implement this by using the output vector of the high level to inhibit the irrelevant state elements in the input to the lower level. The low level will then learn a mapping from that reduced set of states to the output actions. This model also contains a pseudoreward interaction, so the lower level is rewarded for responding according to the rule output by the upper level (if the upper level does not output a rule then the lower level just receives the environmental reward).

#### 5.3.2 Performance

**Fig 12:**
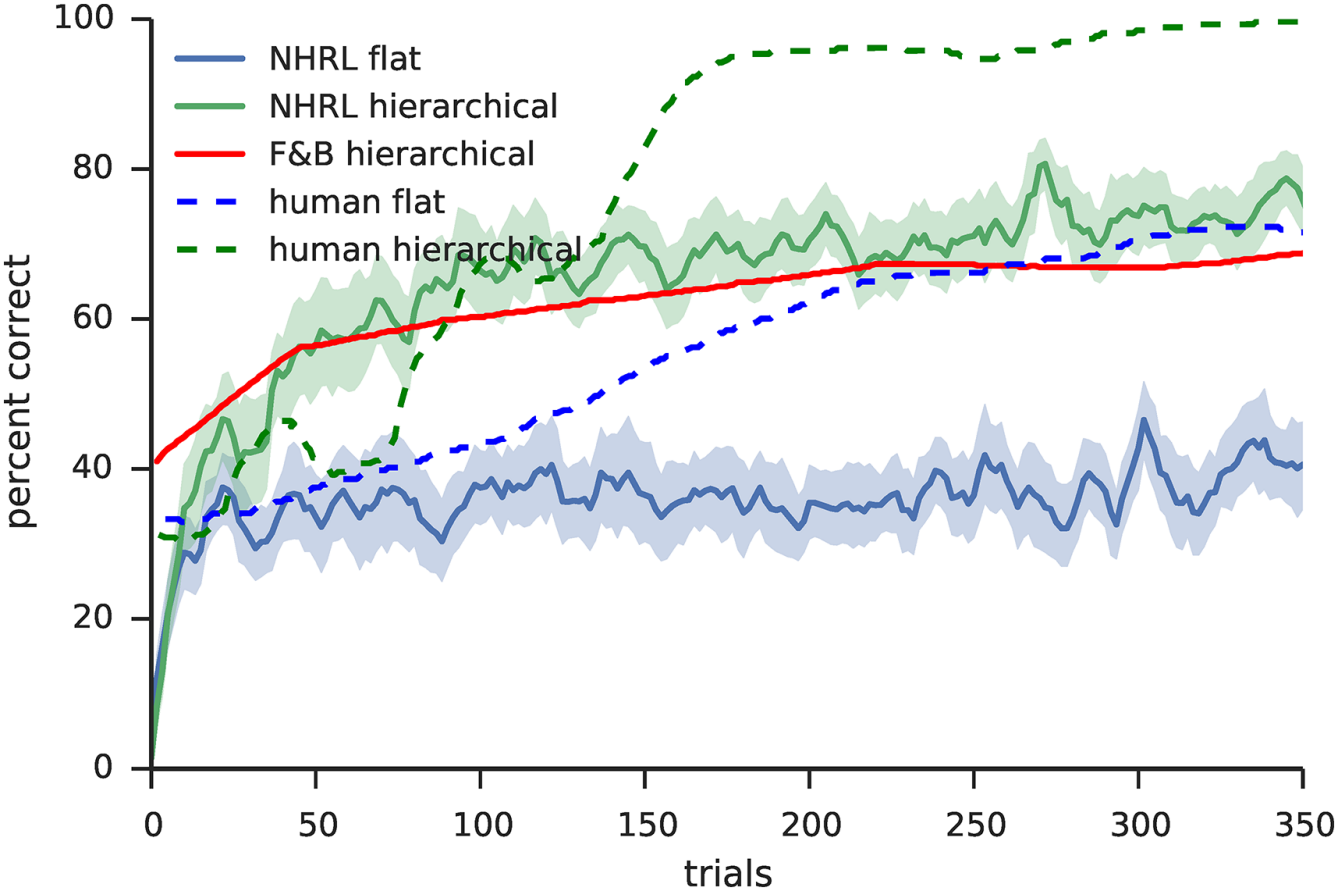
Performance of the NHRL model on the hierarchical stimuli task. Relative to human data from [59] as well as model data from [18] (performance of [18] on flat condition not reported).

**Fig 13:**
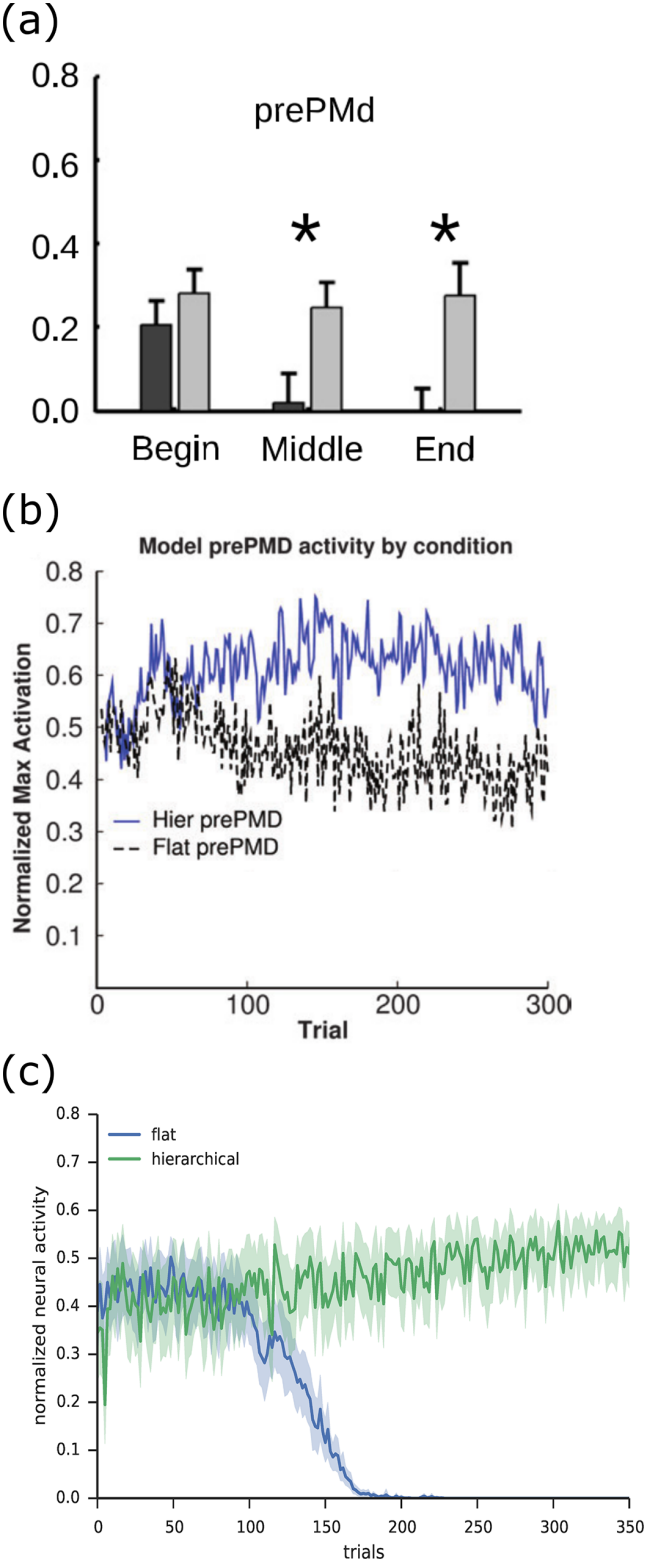
Neural activation on the hierarchical stimuli task. (a) fMRI activation during the flat (dark grey) and hierarchical (light grey) conditions, relative to baseline, from [59]. Showing activity in dorsal pre-premotor cortex (the region found to be associated with higher level hierarchical reasoning). Averaging across trials 0–120, 120–240, and 240–360 in the Begin, Middle, and End groups, respectively. (b) Normalized neural activity of high level output population from [18]. (c) Normalized neural activity of high level output population in NHRL model.

Fig 12 shows the behavioural results from the model. The *x*-axis shows the number of trials, where each trial is one stimulus presentation, and the *y*-axis shows the percentage of trials answered correctly over time. The figure shows human data from [59] on the flat and hierarchical conditions. Note that this is the performance of the single subject “closest to the group mean” in each condition; only terminal accuracy was reported for the group mean—84% in the hierarchical condition and 58% in the flat condition. Fig 12 also shows the performance of the model from [18] on the hierarchical task (performance on the flat condition was not reported), as well as the performance of the NHRL model. Note that the NHRL model is the same in the flat and hierarchical conditions; in the previous experiment we kept the task the same and changed the model to be either flat or hierarchical, whereas here we keep the model the same and change the task to be either flat or hierarchical. The only difference between the model in the flat and hierarchical condition is the scale on the bias current injected into the state population of the action values component (Fig 2). This was a free parameter fit to the neural data from [59] (see Fig 13).

The first observation is that neither our model nor that of [18] achieves human level performance in the hierarchical condition. Performance peaks at around 70% accuracy, below the 84% group mean. However, these results do demonstrate the advantage of hierarchical processing, in that, as with humans, our model is able to learn more quickly on the hierarchical version of the task. Thus we can see the benefit of incorporating hierarchical reasoning in neural models, while still observing that there is more work to be done.

The main neural result from [59] was that activity in anterior prefrontal regions was greater in the hierarchical versus the flat condition. This is consistent with the mapping we established previously. However, the authors found a particular temporal pattern of activation in those anterior regions, which provides a more detailed point of comparison. Namely, both conditions began with the same high activity level, and then the activity decreased in the flat condition. *A priori* it might seem equally likely that both conditions begin with a low activity level that then increases in the hierarchical condition; thus this result provides a unique, testable signature of hierarchical processing in the brain.

Fig 13 shows the comparison between the flat and hierarchical activation in the human data [59], model from [18], and the NHRL model. It can be seen that the NHRL model shows initially equal activity in both conditions, followed by a decrease in the flat condition at the same time scale as the human data. Quantitatively comparing this result to the human data is difficult, as they represent very different types of data (changes in fMRI activation versus absolute neural activity). Similarly, we cannot say whether our model provides a better or worse fit than that in [18]. The important point is that the model captures the observed trend of a decrease in the flat condition, rather than an increase in the hierarchical condition. In other words, it is at least as plausible as the model in [18] in this respect, while providing all the functional advantages described previously (e.g., full temporally extended reinforcement learning).

The fact that the model is able to recreate this effect supports its biological plausibility. However, one of the main advantages of creating functional neural models is that we can find mechanistic explanations for the observed phenomena, rather than simply matching the data. Thus it is interesting to explore *why* the model produces this activity pattern.

To begin, we can examine why the output activity is high or low. The activity levels are due to the output vectors associated with the different actions of the high level. Recall that these vectors are used to inhibit the state input to the lower level. In the flat condition no state abstraction is possible, so the output vector should be zero. However, in the NEF there is no necessary connection between the represented value and the neural activity levels; that is, it is not obvious why a zero valued vector should result in low spiking activity. The key is that in these inhibitory circuits it is important that the inhibition signal be a very exact representation of zero (when not trying to inhibit). Otherwise small fluctuations around zero are magnified in the inhibited signal, introducing unwanted noise. A more accurate zero can be represented by aligning all the firing intercepts of the neurons with the zero value. This has the functional effect of a cleaner inhibition signal, but it also has the neural effect of linking the represented value to the neural firing rate. Thus this functional optimization explains why we find lower activity in the flat condition.

The initial tendency to a high activity level is due to a hypothesis we have included in this task—that the brain has a preference for rule-based explanations. Specifically, we added a small (10%) positive bias to the reward for the high level when it chose one of the two rules as the output action, even if it was the wrong rule; in other words, the reward for the high level was ±1.5 if it selected the null action, but -1.35/+1.65 if it selected the shape or orientation rule. This makes it more likely that the model will pick the non-null actions during the initial exploration phase, meaning it will have higher output activity. Eventually the model will learn the correct response (+1.5 is still better than -1.35), which will then lead to a decrease in the activity levels; the bias just serves as an initial nudge in the hierarchical direction. This modification represents a specific prediction/hypothesis: the brain has a small positive reward bias associated with learning abstract rules. We will discuss how this might be tested empirically in Section 6.3.

## 6 Discussion

### 6.1 Model extensions

In this work we have presented the first model to perform hierarchical reinforcement learning in a biologically detailed neural simulation. However, this work is only an initial step in trying to model how hierarchical reinforcement learning might be implemented in the brain. There are many ways in which it could be improved or extended; here we will focus on two of the most critical, namely model-based reasoning and learning hierarchical structure.

All the methods used in this work have been model-free, meaning that the agent does not have an explicit model of its environment. Model-based methods have many potential strengths, such as a more efficient use of environmental samples, and are certainly an important part of the brain’s RL processing [67]. For example, model-based reasoning might explain the gap between human and simulation performance on the hierarchical stimuli task.

Although model-based approaches to HRL are relatively unexplored, [21] discusses how model-based methods can be applied in the options framework (and their points apply in general to any SMDP-based approach). The key is to learn a model of each abstract action, rather than modelling the overall transition/reward function. An action model encodes the reward and terminal state to be expected when selecting that action in any given state. The agent can use that model to internally simulate the effect of selecting an abstract action, either to generate a simulated TD update or as part of a planning process. Adding this type of reasoning into a neural model would be quite complex, but would be an important complement to the model-free RL presented here.

The second open problem this model highlights is the autonomous learning of hierarchical structure. In this model we have assumed that the hierarchical structure of the model is defined by the modeller. That is, the modeller decides how the problem is broken down into subtasks, and how the different hierarchical levels interact (by defining the context/state/reward interactions). It would be interesting and important to explore how this model could incorporate more autonomous methods for constructing the hierarchical structure. This is one of the major open problems in HRL, and is an active area of research, especially in regards to how this could occur in a neural system [2, 25, 40, 53, 68].

Interestingly, this issue is closely related to the problem of model-based reasoning, as many approaches to structure learning are based on analyzing a model of the environment (e.g., [69–72]). Thus making progress on one of these issues is likely to lead to progress in the other as well.

As mentioned, there are many other avenues of potential development for this model. We discuss another interesting way to extend this model, through the implementation of recurrent hierarchical connections, in the supplementary material (S1 File).

### 6.2 Experimental comparisons

Another important direction for the model is to continue to apply it to experimental data from studies of hierarchical learning, as we did in Section 5.3. There are two studies in particular that would be interesting to recreate. The first uses a modified version of the delivery task (Section 5.2) in order to search for pseudoreward signals associated with the completion of subtask goals [41]. The second uses a hierarchical bandit task to search for separable, simultaneous error signals in the brain, corresponding to different hierarchical levels [42]. The flexible structure of the NHRL model could be applied to these tasks, allowing us to compare human and model performance at both a behavioural and neural level. This would serve to further support the biological plausibility of this model, as well as generate new understanding of the links between neural computation and behavioural performance in these hierarchical settings.

### 6.3 Model predictions

A great advantage of building mechanistic neural models is their capacity to generate predictions, both to guide future empirical research and to verify the biological plausibility of the model. In this section we present some of the predictions that can be generated from this model.

#### 6.3.1 Dual training system

Previously we described a hypothesized neuroanatomical mapping for the dual training system, and mentioned that while it was plausible it was also rather speculative and untested (Section 4.6). It is useful then to discuss how one might investigate that theory experimentally. The key prediction is that updates in the dorsal striatum (representing the current *Q* function) should be time delayed relative to those in the ventral striatum. For example, imagine a rodent in a T-maze. If an unexpected reward were placed in one arm of the maze, we would expect a positive prediction error in the striatum, as normal. The unique prediction of the dual training system is that the prediction error should appear first in ventral striatum, and only afterwards in dorsal striatum.

The length of the time delay between the updates depends on how quickly the animal updates the previous state information (which we suggested might commonly be represented in OFC). Recall that the current *Q* function can only be updated when the current and stored state are the same, which occurs immediately after the stored state is updated. Thus we would expect to see the following sequence: reward received, prediction error in ventral striatum, activity change in OFC (not necessarily correlated with prediction error), and finally prediction error in dorsal striatum. A more dramatic delay could be observed if the animal were moved out of the rewarded state before the OFC update occurred. Then we would not expect a prediction error in dorsal striatum until the next time the animal reaches the rewarded state. However, given the rapidity of the OFC updates, it would likely be difficult to interrupt the update sequence in this way.

#### 6.3.2 Integrative discount

Another unique aspect of this model is the integrative discount mechanism. The key signature of the integrative discount would be a neural representation that changes over the course of action execution. More specifically, the model predicts that the rate of change should be proportional to the value of the previously selected action. Previous work has shown that striatal neurons systematically respond to the passage of time during task delay periods [73]. In addition, [73] showed that the rate of change scaled with the length of the delay period. Unfortunately, in that study the action value was tied to the temporal delay (executing the action becomes less rewarding because the animal has to wait a longer time to receive reward), so we cannot distinguish whether the rate of change was driven by the action value or the length of the delay. In order to separate these two we would need to vary the magnitude of the reward independently of the length of the delay period. Our model predicts that there would be striatal neurons who rescale their temporal firing patterns according to the value of the reward, regardless of the length of the delay.

#### 6.3.3 Rule-based explanation bias

Another prediction of the model, and one that could be tested in human subjects, arose in relation to the hierarchical stimuli task from [59]. Namely, the model predicts that the initial propensity of the hierarchical processing region (identified in pre-premotor cortex) to a high activity level is due to an internally generated bias applied to the reward signal. Experimentally, this would appear as stronger positive prediction errors and weaker negative prediction errors on the hierarchical version of the task relative to the flat version. This could be used to create a measure of the bias in each subject, for example by calculating the ratio between the average positive prediction error in the hierarchical and flat scenarios. The model would then predict that the bias measure would correlate with the magnitude of the high activity bump in the pre-premotor region in the flat condition. Specifically, the bias should be correlated with the width of the bump—the stronger the subject’s bias, the longer they should persist in trying to find a hierarchical rule.

## 7 Conclusion

In this work we have presented the first model to provide a detailed neural implementation of hierarchical RL. This model is able to perform HRL while incorporating important constraints of a biological system, such as local information transfer, continuous environments, temporally extended action sequences, and noisy/heterogeneous/imprecise components. By overcoming the challenges of these more general environments, the NHRL model brings us closer to understanding the complex performance of real brains, for which these challenges are the norm. More specifically, this provides important evidence that the abstract computations of HRL can be adapted so as to be plausibly implemented in real brains.

We have analyzed the model’s performance across a range of tasks, including flat and hierarchical navigation and a hierarchical stimuli–response task. While these tasks are still vastly simplified relative to those we easily navigate in everyday life, they demonstrate the advantages of a hierarchical model, in terms of functional performance and accounting for neurophysiological data.

Along with all of the positive outcomes outlined above, it is important to emphasize that this model has many avenues for further improvement. Processes such as model-based reasoning and the autonomous learning of hierarchical structure are key aspects of the hierarchical story, but absent from this model. Work in those directions would greatly expand the functional and predictive power of the model, and bring us closer to understanding the full range of the brain’s reinforcement learning ability.

## Supporting information

S1 File Appendix. Includes comparisons to other HRL approaches, demonstrations of performance for individual components within the model, and a proof for the theoretical convergence of our modified discounting mechanism.
(PDF)
