# Supplementary material for: A neural model of hierarchical reinforcement learning
Source: PLoS One. 2017 Jul 6;12(7):e0180234. doi: 10.1371/journal.pone.0180234 (PMC5500327; doi:10.1371/journal.pone.0180234)
Supplement: S1 File — Includes comparisons to other HRL approaches, demonstrations of performance for individual components within the model, and a proof for the theoretical convergence of our modified discounting mechanism. (PDF) [file pone.0180234.s001.pdf]

A neural model of hierarchical reinforcement learning: Supporting information

1 Relation to other HRL approaches

It is helpful to compare the hierarchical mechanisms of this model to the various computational approaches to HRL (e.g., [1–3]), as another way of elucidating the features of this model. Firstly, it is important to note that this model is not an exact recreation of any of those theories—the process of neural implementation requires many adaptations and modifications, as we have seen in previous sections. In addition, the hierarchical structure of this model is not fixed, and can combine aspects of the various HRL frameworks depending on how the hierarchical interactions are constructed.

Rather than recreating any particular HRL approach, the goal in the hierarchical structure of this model was to preserve, as far as possible, the basic operation of the flat SMDP model. As it happens, that was also the goal of the authors of the "options" HRL framework [2]. As they put it, "What is the minimal extension of the reinforcement learning framework that allows a general treatment of temporally abstract knowledge and action?" ([2], pg. 182). For that reason, this model bears the strongest similarity to the options framework; for example, the SMDP learning update (Eq. 3 in main text) is almost identical to the options approach.

1.1 Recurrent hierarchical structure

One important way in which this model differs from the options framework, and is more similar to the MAXQ framework of [3], is that the actions are placed into a fixed hierarchical structure. In the options framework an option's policy is defined over all the available actions in a given state. In this model the actions are spread across different layers, and each policy is only defined across the actions of that layer.

However, it would be possible to implement the flexible structure of the options framework using essentially the same basic SMDP model (see Figure A). The key would be to recursively connect the output of the model to its own input; the hierarchical interactions would be the same (context, state, and reward), but instead of, e.g., the output action in the context interaction modifying the state of a lower level, it would modify its own state input. This would allow the model to represent policies with potentially infinite hierarchical depth, as in the options framework.

More generally, this relates to an important issue in the study of hierarchical reasoning in the brain, which is that hierarchical computation/behaviour need not be implemented by hierarchical neuroanatomical structure [4, 5]. Another way of stating this is that behaviour at different temporal scales does not need to be represented in distinct regions in the brain — recurrent connections allow the same network of neurons to represent information across different temporal scales through recurrent temporal dynamics. The above architecture would be an illustration of that point for the case of HRL — an implementation of hierarchical reinforcement learning without hierarchical structure.

The main challenge in this approach is that the recursive model needs to explicitly keep track of the "call stack"—the record of previously selected abstract actions. This

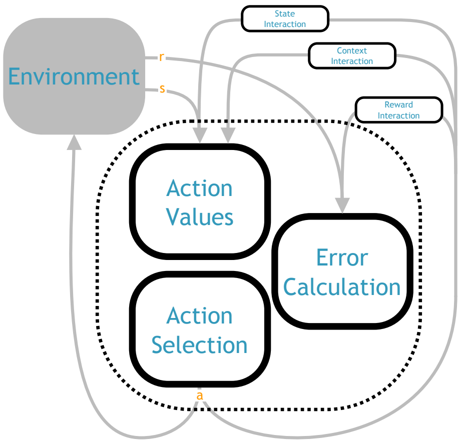

Fig A. Example hierarchical architecture using a recursive structure (compare to Fig. 5 in the main text).

is necessary so that when an action terminates the agent can be returned to the appropriate internal state. This kind of detailed control and timing is difficult to implement in a neural model, although there are NEF models that demonstrate similar behaviour (e.g., [6]). In contrast, with a fixed hierarchical structure the "stack" is implicit in the hierarchy itself.

However, it is important to note that these options are not mutually exclusive. The brain could, and almost certainly does, employ a mixture of recursive and hierarchical approaches. In particular, the disadvantage of a fixed hierarchy is that it imposes a fixed limit on the hierarchical depth—if a system has two layers, then it can only learn at two different levels of abstraction. One reasonable hypothesis would be that lower levels of RL processing are distributed across separate hierarchical layers, while the top levels of the hierarchy contain the recursive connections that allow for processing of arbitrary hierarchical depth. The slower, long term reasoning of the higher levels would be more suited to the serial processing, while the quick low level decisions could proceed in parallel.

2 Performance of individual components

In this section we demonstrate the internal operation of various key components of the model, in order to illustrate the functions they perform.

Figure B shows the output of the action values component. At the left we can see the input state; in this case, these are simulated place cell activations, with each line representing the activity of one cell. As the agent moves through the state space different cells become active. The middle figure shows the activity of the neurons representing the current Q function; each row corresponds to one neuron, and each dot is a spike from that neuron. On the far right is the vector $Q(s)$ that is decoded from the output of those neurons. We include the neural activities just to emphasize that all of the functions we describe here are being performed via neural computations. In future figures we will just show the decoded values, as they contain the meaningful, interpretable information.

In this case the agent is approaching a rewarded state so, as expected, the action values are ramping up. The target is to the south east of the agent, so those actions have the two highest values, and we can see that as the agent moves eastward eventually the value of moving south becomes dominant. The actions that move away from the target (north and west) continue to have a low value throughout. In other

words, the component output matches the Q values we would expect in the given state.

Figure C demonstrates the activity of the dual training system. At the top left we can see the previous and current state. To the right of that is the output of the population computing the thresholded distance between the two states; note that whenever the previous state is updated there is a brief window when the current and previous state are close to each other, and then they progressively diverge, as we would expect. The principle of the dual training system is that whenever the states are the same the output of the Q functions should be the same. Below the states we can see the output of the previous and current Q function, and the output of the population computing the difference between them. This population is inhibited by the state difference, and we can see that its output is zero whenever the state distance exceeds the threshold. The computed value difference is then used to update the current Q function, which can be observed in the deviations in the (e) signals (although this is complicated by the fact that the input state is also changing).

Figure D demonstrates the performance of the action selection component. To the left we can see the input to the component, a set of action values $Q(s)$. In the middle is the output of the basal ganglia network. Note that it has selected the highest valued action, but the selection is somewhat noisy and the output is inverted (the selected element has the lowest value). The rightmost figure shows the output of the final population in the action selection component, which has the the same action selected but with a much cleaner output of 1 for the selected action and 0 elsewhere.

Figure E shows the SMDP TD error calculation process. The inputs of the error calculation are shown on the top: the previous action value ($Q(s, a)$), reward (r), and current action value ($Q(s', a')$). The bottom left shows the integrative discount, which is the integrated value of the previous action. Since $Q(s, a)$ is constant, the discount signal is a linear ramp. To the right is the integrated reward; it begins at zero, integrates the received reward from $t = 0.2$ to $t = 0.5$, and then remains at the resulting value since there is no more reward. In the bottom right is the calculated SMDP TD error, a continuous implementation of Eq. 12 in the main text. Initially the prediction error is zero, as the current and previous state have the same value and there is no reward. Then the agent receives some reward, which generates a positive prediction error. At $t = 0.7$ the current action value increases, further increasing the prediction error for the previous action value. We can also see the effect of the discount, reducing the future value over time.

Finally, in Figure F we can see the calculated error signal being applied to the Q value representation. The continuous SMDP TD error signal is shown on the left; this is the output signal from the network shown in Figure E, but with realistic inputs. Recall that the SMDP TD error is disinhibited by the action termination signal, shown in Figure F(b). It can be seen in Figure F(c) that the error is gated by this termination signal—it is only non-zero when the action terminates. As expected, when the error signal is applied the value of the previously selected action increases or decreases, proportionate to the error value. Thus this implements the TD update (Eq. 3 in main text).

3 Convergence of modified discounting method

One potential concern with the modified discount mechanism described in Section 5.4 is that we lose the theoretical convergence guarantees that are one of the strengths of TD learning. In this section we show that our approach is essentially equivalent in terms of theoretical convergence, and the same proofs apply to our integrative discount method.

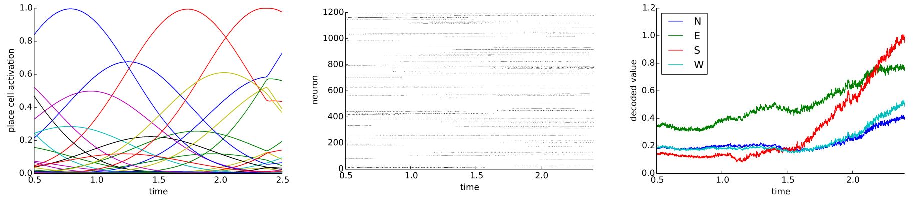

Fig B. Example input and output for the action values component. The input consists of simulated place cell activations, and the output (showing spikes and decoded values) indicates the value of moving in the four cardinal directions in a spatial navigation task (the target is to the south east of the agent).

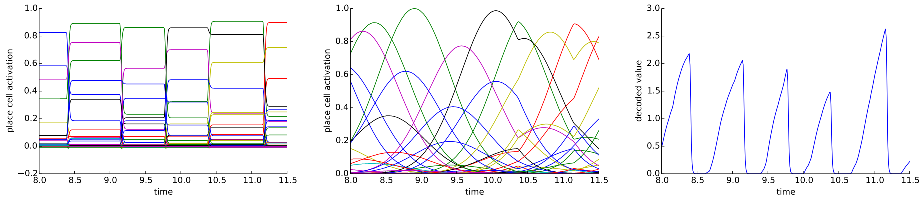

(a) Previous state **(b)** Current state **(c)** State difference

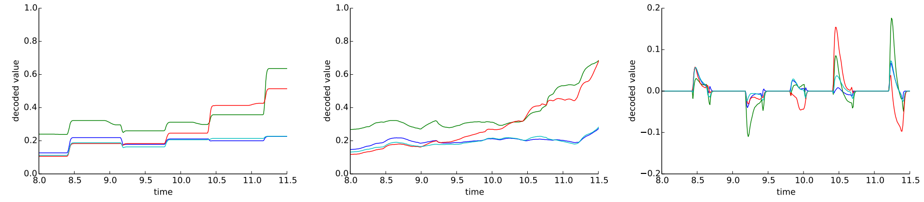

(d) Previous state Q values **(e)** Current state Q values **(f)** Value difference

Fig C. Computations of the dual training system. The previous and current state are shown in (a) and (b), respectively. (c) shows the output of the population computing the thresholded distance between those states. (d) and (e) show the Q values of the previous and current state. (f) shows the difference between those values, which is inhibited by (c) and used as the training signal for (e).

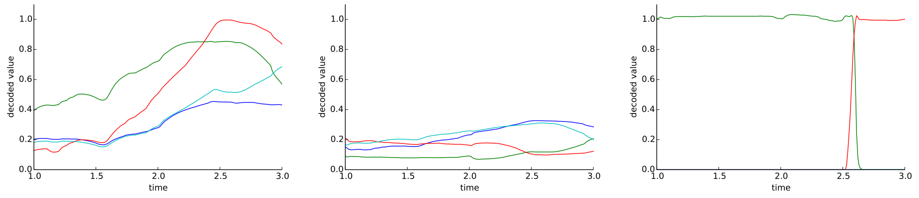

(a) **(b)** **(c)**

Fig D. Operation of the action selection component. Showing (a) input action values, (b) output of the basal ganglia circuit model, and (c) the final thalamic model output, indicating 1 for the selected action and 0 elsewhere.

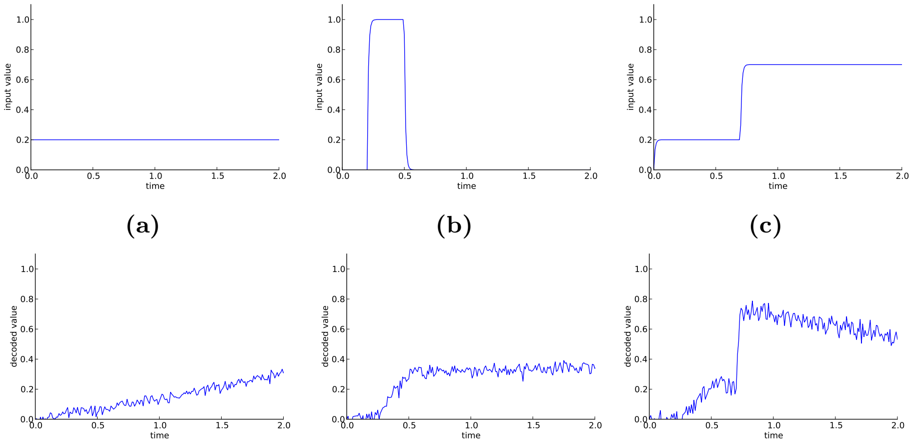

(a) (b) (c)

(d) (e) (f)

Fig E. Computations of the error calculation network. Example input signals are shown in (a), (b), and (c) (the previous action value, reward, and current action value, respectively). (d) shows the integrative discount, (e) shows the accumulated reward, and (f) shows the overall output of the error calculation. Observe that (f) is equal to (c) + (e) - (d) - (a) (see Eq. 12 in main text).

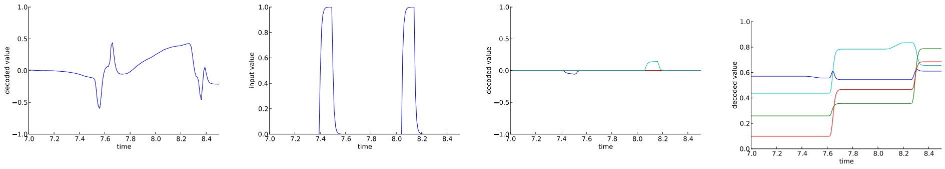

(a) Continuous error signal **(b)** Termination signal **(c)** Gated error signal **(d)** $Q(s)$ values

Fig F. Example application of the error signal to update Q values. (a) shows the error signal, analogous to that shown in Figure E. (b) shows the action termination signal, which is used to gate the signal in (a), resulting in (c). (d) shows the Q values, demonstrating the impact of the error update on the represented values.

For convenience we restate the Q-value update from Eq. 12:

$$\delta(s,a) = Q(s',a') - Q(s,a) - \tau\gamma Q(s,a) + \sum_{i=0}^{\tau-1} r_i \tag{1}$$

To make the notation more precise, we rewrite Equation 1:

$$\delta_t(s_t,a_t) = Q_t(s_{t+1},a_{t+1}) - Q_t(s_t,a_t) - \gamma\tau(s_t,a_t)Q_t(s_t,a_t) + \sum_{i=0}^{\tau(s_t,a_t)-1} r_i(s_t,a_t) \tag{2}$$

having made the following substitutions:

$$\begin{aligned}
Q_t &\leftarrow Q\\
\delta_t &\leftarrow \delta\\
s_t &\leftarrow s\\
a_t &\leftarrow a\\
s_{t+1} &\leftarrow s'\\
a_{t+1} &\leftarrow a'\\
\tau(s_t,a_t) &\leftarrow \tau\\
r_i(s_t,a_t) &\leftarrow r_i
\end{aligned}$$

Now define:

$$\begin{aligned}
\lambda(s_t,a_t) &\stackrel{def}{=} 1 + \gamma\tau(s_t,a_t)\\
\Upsilon(s_t,a_t) &\stackrel{def}{=} \sum_{i=0}^{\tau(s_t,a_t)-1} r_i(s_t,a_t)\\
\bar{r}(s_t,a_t) &\stackrel{def}{=} \Upsilon(s_t,a_t)/\lambda(s_t,a_t)\\
\bar{\gamma}(s_t,a_t) &\stackrel{def}{=} 1/\lambda(s_t,a_t)\\
\bar{\alpha}_t(s_t,a_t) &\stackrel{def}{=} \alpha_t(s_t,a_t)\lambda(s_t,a_t)
\end{aligned}$$

We may understand λ as a normalization factor, and Υ as the probabilistic reward obtained from selecting action a while in state s after a delay period containing τ sub-rewards r_i. We assume that $\tau(s_t,a_t)$ and $r_i(s_t,a_t)$ are random samples that depend only on s_t and a_t.

Now we may rearrange Eq. 3 from the text using the above equations with a learning rate of $\alpha_t(s_t,a_t)$:

$$\begin{aligned}
Q_{t+1}(s_t,a_t) &= Q_t(s_t,a_t) + \alpha_t(s_t,a_t)\delta_t(s_t,a_t)\\
&= (1-\alpha_t(s_t,a_t)\lambda(s_t,a_t))Q_t(s_t,a_t) + \alpha_t(s_t,a_t)\left[\Upsilon(s_t,a_t) + Q_t(s_{t+1},a_{t+1})\right]\\
&= (1-\bar{\alpha}_t(s_t,a_t))Q_t(s_t,a_t) + \bar{\alpha}_t(s_t,a_t)\left[\bar{r}(s_t,a_t) + \bar{\gamma}(s_t,a_t)Q_t(s_{t+1},a_{t+1})\right]
\end{aligned} \tag{3}$$

The update rule (Equation 3) is now in the standard form for $\texttt{SARSA}(0)$ [7] (see also Equation 3 from [8]) with respect to the reward $\bar{r}$, learning rate $\bar{\alpha}$, and discount factor $\bar{\gamma}$. To show convergence, we make the same set of assumptions from [8]:

1. The policy π is "greedy in the limit with infinite exploration" (GLIE); every state-action pair is explored infinitely often, and π becomes greedy as $t \to \infty$ with probability 1.

2. The Q-values are represented and updated perfectly using a lookup-table representation.

The first assumption is satisfied if the MDP is "communicating" (i.e., there exists a path between any two states with non-zero probability), and for a large class of non-stationary policies (e.g., ϵ-greedy with appropriately decaying ϵ) [7]. There is also strong evidence that the second assumption can be relaxed to allow for a representation that is a linear combination of neural basis functions [9, 10].

To satisfy the remaining requirements of Theorem 1 from [8] we need the following Lemma which relates conditions on the parameters from the chosen algorithm to those from the effective $\texttt{SARSA}(0)$ implementation:

Lemma. *If there exists some finite K such that $1 < \lambda(s_t, a_t) \leq K$ and $0 \leq \alpha_t(s_t, a_t) \leq 1/\lambda(s_t, a_t) < 1$, and for every state s and action a we have:*

- $\sum_t \alpha_t(s, a) = \infty$
- $\sum_t \alpha_t^2(s, a) < \infty$
- $\alpha_t(s, a) = 0$ *if* $s \neq s_t$ *or* $a \neq a_t$
- $\mathit{Var}\{\Upsilon(s, a)\} < \infty$

then we satisfy the remaining conditions of Theorem 1 from [8], in particular $0 < \bar{\gamma}(s_t, a_t) < 1$, $0 \leq \bar{\alpha}_t(s_t, a_t) \leq 1$, and for every state s and action a we have:

- $\sum_t \bar{\alpha}_t(s, a) = \infty$
- $\sum_t \bar{\alpha}_t^2(s, a) < \infty$
- $\bar{\alpha}_t(s, a) = 0$ *if* $s \neq s_t$ *or* $a \neq a_t$
- $\mathit{Var}\{\bar{r}(s, a)\} < \infty$

Proof. Since $\bar{\gamma}(s_t, a_t) = 1/\lambda(s_t, a_t)$ and $1 < \lambda(s_t, a_t) \leq K$ is finite, we have $0 < 1/K \leq \bar{\gamma}(s_t, a_t) < 1$. Similarly, since $\bar{\alpha}_t(s_t, a_t) = \alpha_t(s_t, a_t)\lambda(s_t, a_t)$ and $0 \leq \alpha_t(s_t, a_t) \leq 1/\lambda(s_t, a_t)$, we have $0 \leq \bar{\alpha}_t(s_t, a_t) \leq 1$. Then since $\bar{r}(s_t, a_t) = \Upsilon(s_t, a_t)/\lambda(s_t, a_t)$ and $\lambda(s_t, a_t) > 1$, $\mathrm{Var}\{\bar{r}(s, a)\} < \infty$. Finally $\sum_t \bar{\alpha}_t(s, a) > \sum_t \alpha_t(s, a) = \infty$, and similarly $\sum_t \bar{\alpha}_t^2(s, a) \leq K^2 \sum_t \alpha_t^2(s, a) < \infty$ since K is finite, and $\bar{\alpha}_t(s, a) = 0$ unless $s = s_t$ and $a = a_t$. □

We remark that these requirements are met if, for instance, $0 < \tau \leq D$ gives a finite upper-bound on the maximum possible time-delay, $\gamma > 0$, and $0 \leq \alpha \leq 1/(1 + \gamma D)$, with a learning rate that approaches $\Theta(1/t)$ (by the divergence of $\int_1^\infty 1/t \, dt$ and the convergence of $\int_1^\infty 1/t^2 \, dt$), and assuming bounded sub-rewards (by Popoviciu's inequality applied to $|\Upsilon| \leq D \cdot \max |r_i|$).

Then Q_t converges as $t \to \infty$ by Theorem 1 from [8] applied to Equation 3 with a slight modification to allow for a probabilistic γ that depends on s and a. Since $0 < E\{\bar{\gamma}(s, a)\} < 1$ and $\mathrm{Var}\{\bar{\gamma}(s, a)\} < 1$, and $\bar{\gamma}(s_t, a_t) \perp\!\!\!\perp Q_t(s_{t+1}, \cdot)$, the proof of Theorem 1 still holds by satisfying Lemma 1 [8] with respect to our modified update operator.

References

1. Parr R, Russell SJ. Reinforcement learning with hierarchies of machines. In: Advances in Neural Information Processing Systems; 1998.
2. Sutton RS, Precup D, Singh S. Between MDPs and semi-MDPs: A framework for temporal abstraction in reinforcement learning. Artificial Intelligence. 1999;112(1-2):181–211.
3. Dietterich T. Hierarchical reinforcement learning with the MAXQ value function decomposition. Journal of Artificial Intelligence Research. 2000;13:227–303.
4. Botvinick MM, Plaut D. Doing without schema hierarchies: A connectionist approach to routine sequential action and its pathology. Psychological Review. 2004;.
5. Uithol S, van Rooij I, Bekkering H, Haselager P. Hierarchies in action and motor control. Journal of Cognitive Neuroscience. 2012;24(5):1077–1086.
6. Eliasmith C, Stewart TC, Choo X, Bekolay T, DeWolf T, Tang Y, et al. A large-scale model of the functioning brain. Science. 2012;338(6111):1202–1205.
7. Rummery G, Niranjan M. On-line Q-learning using connectionist systems. Cambridge University; 1994. September.
8. Singh S, Jaakkola T, Littman ML, Szepesvari C. Convergence results for single-step on-policy reinforcement-learning algorithms. Machine Learning. 2000;39:287–308.
9. Tsitsiklis JN, Van Roy B. An analysis of temporal-difference learning with function approximation. IEEE Transactions on Automatic Control. 1997;42(5):674–690.
10. Melo FS, Meyn SP, Ribeiro MI. An analysis of reinforcement learning with function approximation. In: Proceedings of the 25th International Conference on Machine Learning. New York: ACM; 2008. p. 664–671.
